# Cysteine Dioxygenase 1 Is a Tumor Suppressor Gene Silenced by Promoter Methylation in Multiple Human Cancers

**DOI:** 10.1371/journal.pone.0044951

**Published:** 2012-09-27

**Authors:** Mariana Brait, Shizhang Ling, Jatin K. Nagpal, Xiaofei Chang, Hannah Lui Park, Juna Lee, Jun Okamura, Keishi Yamashita, David Sidransky, Myoung Sook Kim

**Affiliations:** 1 Department of Otolaryngology, Head and Neck Cancer Research Division, The Johns Hopkins University School of Medicine, Baltimore, Maryland, United States of America; 2 Clinical Research Coordination, Instituto Nacional de Câncer (INCA) - Brazilian National Cancer Institute, Rio de Janeiro, Rio de Janeiro, Brazil; 3 Department of Pathology, The Johns Hopkins University School of Medicine, Baltimore, Maryland, United States of America; 4 Department of Epidemiology, University of California Irvine, Irvine, California, United States of America; 5 Department of Surgery, Kitasato University Hospital, Sagamihara, Kanagawa, Japan; University of Hong Kong, Hong Kong

## Abstract

The human *cysteine dioxygenase 1* (*CDO1*) gene is a non-heme structured, iron-containing metalloenzyme involved in the conversion of cysteine to cysteine sulfinate, and plays a key role in taurine biosynthesis. In our search for novel methylated gene promoters, we have analyzed differential RNA expression profiles of colorectal cancer (CRC) cell lines with or without treatment of 5-aza-2′-deoxycytidine. Among the genes identified, the *CDO1* promoter was found to be differentially methylated in primary CRC tissues with high frequency compared to normal colon tissues. In addition, a statistically significant difference in the frequency of *CDO1* promoter methylation was observed between primary normal and tumor tissues derived from breast, esophagus, lung, bladder and stomach. Downregulation of *CDO1* mRNA and protein levels were observed in cancer cell lines and tumors derived from these tissue types. Expression of *CDO1* was tightly controlled by promoter methylation, suggesting that promoter methylation and silencing of *CDO1* may be a common event in human carcinogenesis. Moreover, forced expression of full-length *CDO1* in human cancer cells markedly decreased the tumor cell growth in an *in vitro* cell culture and/or an *in vivo* mouse model, whereas knockdown of *CDO1* increased cell growth in culture. Our data implicate *CDO1* as a novel tumor suppressor gene and a potentially valuable molecular marker for human cancer.

## Introduction

Cancer is caused by aberrant gene regulation, including inactivation of negative regulators of cell proliferation (including tumor suppressor genes; TSG) and activation of positive regulators (such as oncogenes). In addition to genetic alterations involving mutations of oncogenes and TSGs, carcinogenic process can occur through epigenetic changes in gene promoters [1]. Epigenetic changes, heritable changes in gene expression that occur without changes to the DNA sequence, contribute to the development and progression of tumor cells [2] and are considered to be hallmarks of cancer. DNA methylation and histone acetylation are the most frequent and studied epigenetic changes [1]. There are CG-rich regions known as CpG islands that can be located within the 5′end region including promoter, untranslated region and exon 1 of any genes with TSG activity [3]. CpG islands are not usually methylated in normal cells [3], but aberrant hypermethylation in the CpG islands which leads to transcriptional inactivation and gene silencing can be early events in carcinogenesis and is considered to be a common mechanism of loss of TSG function in human cancers [1], [4]. Currently, it is well accepted that epigenetic alterations even predispose to genetic alterations during tumorigenesis [5]. Clinically, TSG methylation can be used as an epigenetic biomarker for tumor diagnosis and prognosis prediction. Thus, knowledge of methylation patterns across the genome can help to identify key TSGs inactivated during tumor formation [6], [7], [8].

Mammalian *cysteine dioxygenase* (*CDO*, EC 1.13.11.20) is a vital enzyme for human health involved in the biodegradation of toxic cysteine and thereby regulation of the cysteine concentration in the body. It is a non-heme structured, iron-containing metalloenzyme involved in the conversion of cysteine to inorganic sulfate, and plays a key role in taurine biosynthesis [9]. The open reading frame of *CDO* gene encodes a protein of 200 amino acids (molecular weight 23 kd, which binds one Fe_2_^−^ ion per molecule). Mouse *cdo* and rat *cdo* have an identical amino acid sequence that is 92% identical to human *CDO*, and most amino acid substitutions are conservative replacements. The *CDO* gene spans about 15 kb and contains 5 exons. The 5′-flanking region of the human *CDO* gene contains several putative consensus *cis*-acting regulatory sequences, in particular for binding of hepatic nuclear factor-3 (HNF-3) and its homologues, that are known to be involved in liver-specific gene transcription. The presence of these consensus binding sites is consistent with the highest level of *CDO* mRNA found in liver extracts compared to other tissue extracts (kidney, lung and brain) [10]. There are two types of CDO; cytosolic (CDO1) and membrane-bound (CDO2), and murine CDO1 has been postulated to be involved in the regulation of protein function and antioxidant defense mechanisms through its ability to oxidize cysteine residues [11].

The human *CDO1* gene is located on chromosome 5 q23.2 which is frequently deleted in advanced lung cancer [12]. Staub et al. [13] assumed that deletion or epigenetic silencing of the chromosomal region 5 q23.2 where *CDO1* is located is a frequent mechanism contributing to colorectal tumorigenesis; *adenomatous polyposis coli* (*APC*) and *mutated in colorectal cancer* (*MCC*) are located in the neighborhood of 5 q23 [12]. It is highly expressed in the liver and placenta, and weakly in the heart, brain and pancreas [14]. Cysteine regulates CDO1 turnover through Ubiqutin-26S proteasome-mediated degradation [15], and a high level of cysteine is cytotoxic and can cause rheumatoid arthritis [16], [17], [18], Parkinson’s disease [17], Alzheimer’s disease [17], increased risk of cardiovascular disease [19] and adverse pregnancy outcomes [20]. Recently, over-expression of CDO1 was described for the Sézary syndrome, an aggressive cutaneous T-cell lymphoma [21].

Previously, we reported a set of candidate genes that comprise part of the emerging “cancer methylome” by using a new promoter structure algorithm and microarray data generated from 22 cancer cell lines derived from 5 major cancer types [22], [23]. In our earlier studies, we examined newly identified cancer-specific methylated genes in a panel of 300 primary tumors representing 13 types of cancer; *oncostatin M receptor-β* (*OSMR*) and *β-1,4-galactosyltransferase-1* (*B4GALT1*) were two of the new genes identified in the study to be methylated in primary CRC tissues but rarely in corresponding normal adjacent mucosa and in non-malignant normal colon tissues [23]. In addition, a combination of pharmacological unmasking and oligonucleotide microarray analysis [24], [25], [26] enabled us to find novel methylated genes in esophageal squamous cell carcinoma (ESCC) and CRC cell lines and primary tissues. *NEFH* [27], *DFNA5* [26], *OSMR* [23], *LIFR* [28] and *NMDAR2B* [25] were representative genes that we found to be epigenetically inactivated in human ESCC and CRC at high frequency. mRNA expressions of these genes in cancer tissues were also significantly down-regulated as compared to normal tissues. Moreover, functional studies suggested tumor suppressive roles for these genes in human CRC and ESCC cell lines.

In this study, we report that *CDO1* was one of the candidate genes identified by the combination of pharmacological unmasking strategy and expression microarray analyses. We observed frequent promoter methylation and downregulation of *CDO1* in multiple types of human cancer. Functional studies revealed that *CDO1* harbors tumor suppressive activity.

## Results

### *CDO1* is Epigenetically Inactivated in Human Colon Cancer

**Figure 1:**
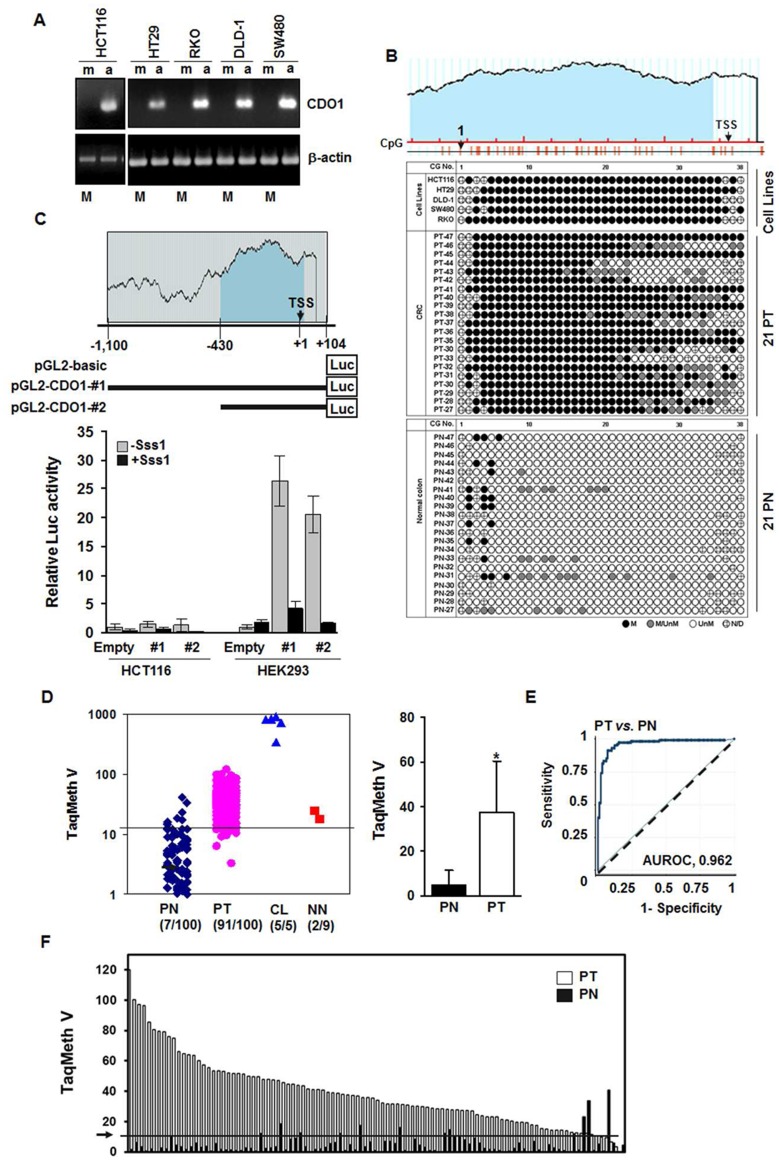
Methylation of the *CDO1* promoter in CRC. **A**, Expression of *CDO1* in CRC cell lines was examined by RT-PCR analysis. No basal expression of *CDO1* was seen in all CRC cell lines, and all these cell lines harbored *CDO1* methylation (M). Silenced *CDO1* was reactivated after treatment with the demethylating agent, 5-Aza-dC, indicating that *CDO1* methylation correlates tightly with loss of gene expression in CRC cell lines. β-actin was used as a loading control. m, cells treated with vehicle only; a, cells treated with 5-Aza-dC (5 µM for three days). L, 1 Kb Plus DNA ladder. **B**, Methylation status of individual CpGs of the island in 5 CRC cell lines and 21 pairs of primary CRC (PT) and their corresponding normal colon tissues (PN) is shown. A total of 38 CGs were numbered from the first to last CG in the sequences as indicated. Black circle, methylation; white circle, unmethylation; grey circle, co-existence of methylated and unmethylated alleles; dashed circle, undetermined. **C**, Analysis of *CDO1* promoter activity by luciferase reporter assay in *CDO1*-negative (HCT116) and -positive (HEK293) cells. The promoter constructs (pGL2-*CDO1*-#1 and -#2) were pre-treated with or without *Sss*I methylase for 8 hrs before transfection. High activity of the *CDO1* promoter was detected in HEK293 where *CDO1* was expressed. Data are expressed as fold increase over pGL2-basic activity. Experiments were done in triplicate, and values indicate means ± SD. Mean values are presented. **D.** Scatter plot of *CDO1* methylation levels in tissues and cell lines (CL) (left). TaqMan methylation values (TaqMeth V) is described in Materials and Method. TaqMan-MSP was performed in duplicate format, and experiments were repeated twice. Data showed reproducible and concordant results. PT, primary CRC; PN, matched normal colon tissues from colon cancer patients; NN, normal colon epithelium from non-cancer patients. Line indicates the optimal cut-off value for *CDO1* calculated from ROC analysis. Sample numbers showing TaqMeth V over the cut-off are indicated. The overall TaqMeth V detected in PT was significantly higher than that in PN (right). TaqMan value of two NN (22%, 2/9) was above the cut-off value (24.56 and 17.86 each), suggesting that a low level of CDO1 methylation can be caused by other unknown mechanisms. **E**, ROC curve analysis of TaqMeth V of *CDO1* in CRC. The Area under the ROC (AUROC) conveys the accuracy in distinguishing matched normal colon (PN) from CRC (PT) in terms of its sensitivity and specificity (*P<0.001*). Solid line, *CDO1*; dashed line, no discrimination. **F**, Methylation levels of normal (PN) and tumor tissues (PT) in individual patients.

In our search for genes epigenetically silenced in human CRC, we performed a combination of pharmacological unmasking and subsequent differential microarray analysis using microarrays containing 22,284 transcripts (Affymetrix) [26]. We used the demethylating agent 5-Aza-2′-deoxycytidine (5-Aza-dC) to reactivate genes epigenetically silenced in three CRC cell lines (HCT116, HT29 and DLD-1). We have previously reported selection criteria for identifying genes frequently inactivated in colon cancer by DNA promoter methylation [26]. We re-analyzed candidate tumor suppressor genes on our gene list and found that the expression of *cysteine dioxygenase 1* (*CDO1*) was ‘absent’ before pharmacological treatment but ‘present’ after 5-Aza-dC treatment in all three CRC cell lines tested. The absent and re-activated *CDO1* expression by 5-Aza-dC in five CRC cell lines (HCT116, HT29, DLD-1, RKO and SW480) were validated by the RT-PCR analysis (**Fig. 1A**).

To investigate whether *CDO1* expression is regulated by promoter methylation, we searched for CpG islands in the *CDO1* promoter by using the online accessible software Methprimer (http://www.urogene.org/methprimer/index1.html). *CDO1* harbors a CpG island in the promoter proximal to the transcription start site (TSS) (**Fig. S1A**). We analyzed methylation status of the CpG island in 5 CRC cell lines (HCT116, HT29, DLD-1, SW480 and RKO) and 4 tumor-adjacent normal appearing tissues (PN) by bisulfite-sequencing. The CpG island was densely methylated in the CRC cell lines but not in normal colon tissues (**Fig. S1B & 1B**). Partial demethylation of the CpG island after 5-Aza-dC treatment in cell lines was also confirmed by bisulfite-sequencing analysis (data not shown). We also performed COBRA in parallel with the bisulfite-sequencing to corroborate *CDO1* methylation in 10 pairs of randomly selected matched tumor (PT) and normal colon tissues (PN) (**Fig. S1C**). The frequency of *CDO1* methylation in tumors (7/10, 70%) was higher than in corresponding normal tissues (1/9, 11.11%). To analyze the *CDO1* methylation status in primary tumors, we examined 21 pairs of PT and PN by bisulfite-sequencing. According to our criteria to determine *CDO1* methylation in cell lines and tissues by bisulfite-sequencing (described in Materials and Methods), the frequency of *CDO1* methylation in tumors was 100% (21/21) and that in normal methylation was 0% (0/21), indicating that *CDO1* is hypermethylated in CRC (**Fig. 1B**). Representative bisulfite-sequencing results of *CDO1* are shown in **Figure S1D**.

To elucidate the role of DNA methylation in the regulation of *CDO1* expression, we made two luciferase reporter constructs (pGL2-*CDO1*-#1 and -#2) containing different portions of the *CDO1* promoter sequences (position −1100 bp to +104 bp for #1, and −430 bp to +104 bp for #2) (**Fig. 1C** **, upper**). Empty pGL2-luciferase plasmids were transfected for mock activity. We transfected these constructs into two cell lines; *CDO1*-negative HCT116 cells and *CDO1*-positive HEK293 cells. Activity of the *CDO1* promoter was minimal in HCT116 cells, but a high level of promoter activity was detected in HEK293 (**Fig. 1C** **, lower**). In addition, both pGL2-*CDO1*-#1 and -#2 constructs had similar levels of *CDO1* promoter activity in HEK293 cells, but induction of CpG methylation with *Sss*I methylase before transfection decreased activity to a minimal level. These results indicate that DNA methylation of the CpG island plays a major role in gene silencing of *CDO1*.

To study promoter methylation of the *CDO1* by quantitative TaqMan-MSP analysis, we designed primers and a probe specifically targeting the CpG island in the *CDO1* promoter (**Fig. S1A**). We increased the sample numbers to 100 pairs of primary CRC (PT) and corresponding normal colon (PN) tissues. Nine normal colon mucosa tissues from non-cancer patients (NN) were included to compare methylation specificity between cancer and non-cancer patients. We also included 5 CRC cell lines that were previously analyzed in the TaqMan-MSP analysis. The distribution of methylation values (TaqMan methylation values, TaqMeth V) exhibit a clear cancer specific pattern; being statistically different between PT and PN (**Fig. 1D** **, left**). Due to heterogeneous clonal patches known to expand beyond the tumor borders, a low level of methylation in PN was also commonly observed. The overall methylation level of *CDO1* detected in PT (38.42±22.97, mean ± SD, n = 100) was significantly higher than those in PN (5.00±6.51, mean ± SD, n = 100) (*P<0.0001, Wilcoxon-Mann-Whitney test*) (**Fig. 1D** **, right**). Methylation of the *CDO1* gene in tissue showed highly discriminative receiver–operator characteristic (ROC) curve profiles, clearly distinguishing PT from PN (**Fig. 1E**). Area under the ROC (AUROC) was 0.962±0.0138 (*P<0.001*). In order to maximize sensitivity and specificity, the optimal cut-off for methylation of the *CDO1* gene was calculated from the ROC analysis (value, 12.50) (PT *vs*. PN). At this cut-off, the optimal specificity was 93% and the sensitivity was 91% (*P<0.001*, *Fisher’s exact* test). In addition to a simple frequency, the comparison of methylation levels of PN and PT from the same individual patients revealed that the majority of PT harbored much higher values than PN (*P<0.0001, Wilcoxon matched-pairs signed-ranks* test) (**Fig. 1F**). A high level of *CDO1* promoter methylation was also found in CRC cell lines (5/5, 100%), consistent with the bisulfite-sequencing and COBRA results. These results indicate that *CDO1* is cancer-specifically methylated in CRC with high frequency.

### *CDO1* is Methylated in Multiple Types of Human Cancer

**Figure 2:**
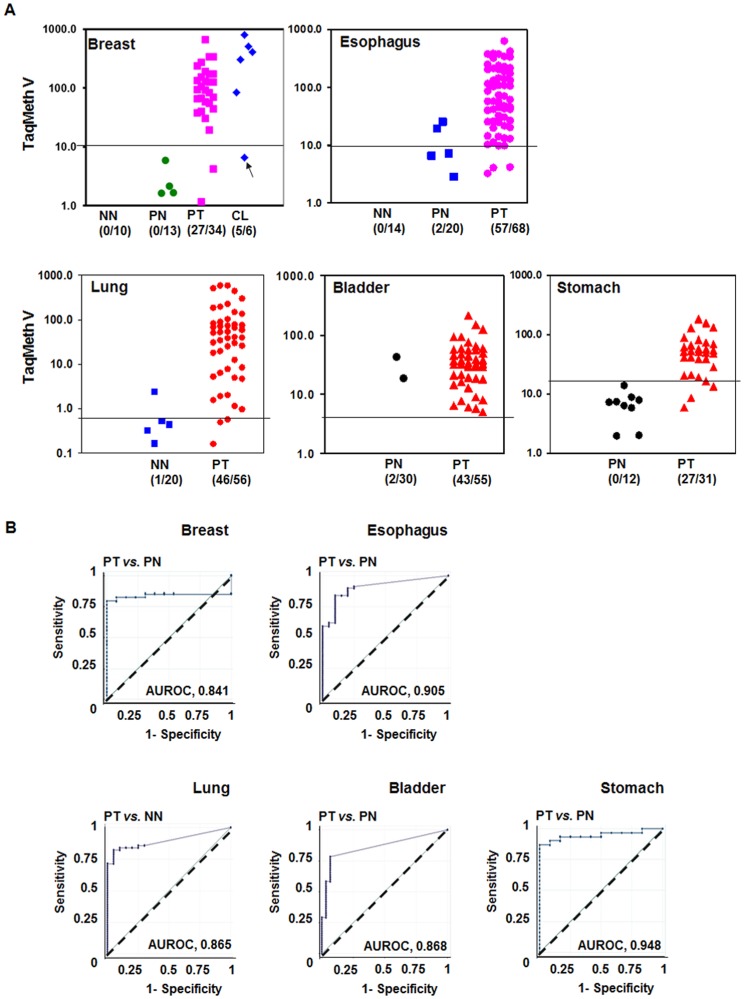
Methylation of the *CDO1* promoter in multiple types of human cancer. A , Quantitative methylation levels of *CDO1* were determined in primary tissues derived from breast, esophagus, lung, bladder, and stomach. TaqMan methylation values (TaqMeth V) is described in Materials and Method. PT, primary tumor; PN, matched normal tissues; NN, normal tissues from non-cancer patients; CL, cell lines. Lines indicate the optimal cut-off value for each tissue. Arrow, MCF-12A, a non-tumorigenic cell line, harbored a low level of *CDO1* methylation (TaqMeth V, 6.2). All assays were performed in duplicate format, and experiments were repeated twice. Data showed reproducible and concordant results. **B**, ROC analysis (PT *vs*. PN) of *CDO1* in multiple human cancers. Solid line, *CDO1*; dashed line, no discrimination.

To investigate the methylation status of *CDO1* in other tissue types, we performed TaqMan-MSP in the *CDO1* promoter in primary tissues derived from breast, esophagus, lung, bladder, and stomach. As shown in **Figure 2A**, the *CDO1* promoter was highly methylated in tumors of all tissue types tested. Breast samples consisted of a non-tumorigenic (MCF-12A) and 5 cancer cell lines (BT20, MDA-MB-231, MCF-7, Hs748.T, Hs578.T), and 3 types of primary tissues (34 PT, 10 PN, and 10 NN). Twenty-seven out of 34 (79%) breast PT harbored values above the optimal cut-off value of 10, and no normal breast tissues from patients with (PN) or without (NN) cancer were above the value (0%). The level of *CDO1* methylation in MCF-12A was below the cut-off. *CDO1* promoter was also highly methylated in bladder cancer. Forty-three out of 55 (78%) bladder PT harbored values above the optimal cut-off value of 5, and only two samples out of 32 (6.2%) normal bladder tissues were above the cut-off value. ROC curve analysis generated the optimal cut-off values (**Fig. 2B**) as well as maximal sensitivity and specificity for each type of cancer. The mean value of TaqMeth V, AUROC, cut-off values, % sensitivity and specificity, and p values of statistical analysis in each type of samples are shown in **Table 1**. The sensitivity and specificity of *CDO1* was over 78% in PT and over 90% in PN, respectively, in all tissue types tested. These results show that *CDO1* is commonly methylated in multiple types of human cancer. The clinicopathological features of the patients analyzed in this study were not available.

**Table 1:** Sensitivity and specificity of CDO1 methylation in multple types of human cancer.

	TaqMeth V								
Tissue	PT	PN	a *P*	AUROC	c *P*	Cut-off	% Sensitivity	% Specificity	d *P*
Colon	38.42±22.97	5.00±6.51	*<0.001*	0.9620±0.013	*<0.001*	12.50	91 (91/100)	93 (93/100)	*<0.001*
Breast	115.61±134.34	0.93±1.69	*<0.001*	0.8416±0.061	*<0.001*	19.23	79 (27/34)	100 (13/13)	*<0.001*
Esophagus	126.12±200.84	3.040±6.95	*<0.001*	0.9056±0.032	*<0.001*	9.75	83 (57/68)	90 (18/20)	*<0.001*
Lung	82.07±141.20	0.05±0.07	*<0.001* b	0.8955±0.033	*<0.001*	0.569	82 (46/56)	95 (19/20)	*<0.001*
Bladder	34.13±40.31	3.04±6.95	*<0.001*	0.8688±0.036	*<0.001*	5.02	78 (43/55)	93 (28/30)	*<0.001*
Stomach	56.93±43.38	5.10±4.13	*<0.001*	0.9489±0.032	*<0.001*	16.57	87 (27/31)	100 (12/12)	*<0.001*

TaqMeth V is expressed as mean ± SD, and TaqMeth V is described in Materials and Methods.

a P value was derived from Wilcoxon-Mann-Whitney test of ^a^PT vs. PN;

b PT vs. NN only in lung.

PT, tumor tissue from cancer patients; PN, normal tissue from cancer patients; NN, normal tissue from non-cancer patients.

AUROC is expressed as mean ± SD, and optimal cut-off values were calculated from ROC analysis.

Methylation level below the cut-offs was considered as unmethylated and over the cut-offs were as methylated.

c P value in ROC analysis;

d P value in Fisher’s exact test.

Sensitivity, positive methylation/total tumor cases; Specificity, negative methylation/total normal cases.

### *CDO1* is Silenced in Cancer Cell Lines and Re-activated by Pharmacological Demethylation

**Figure 3:**
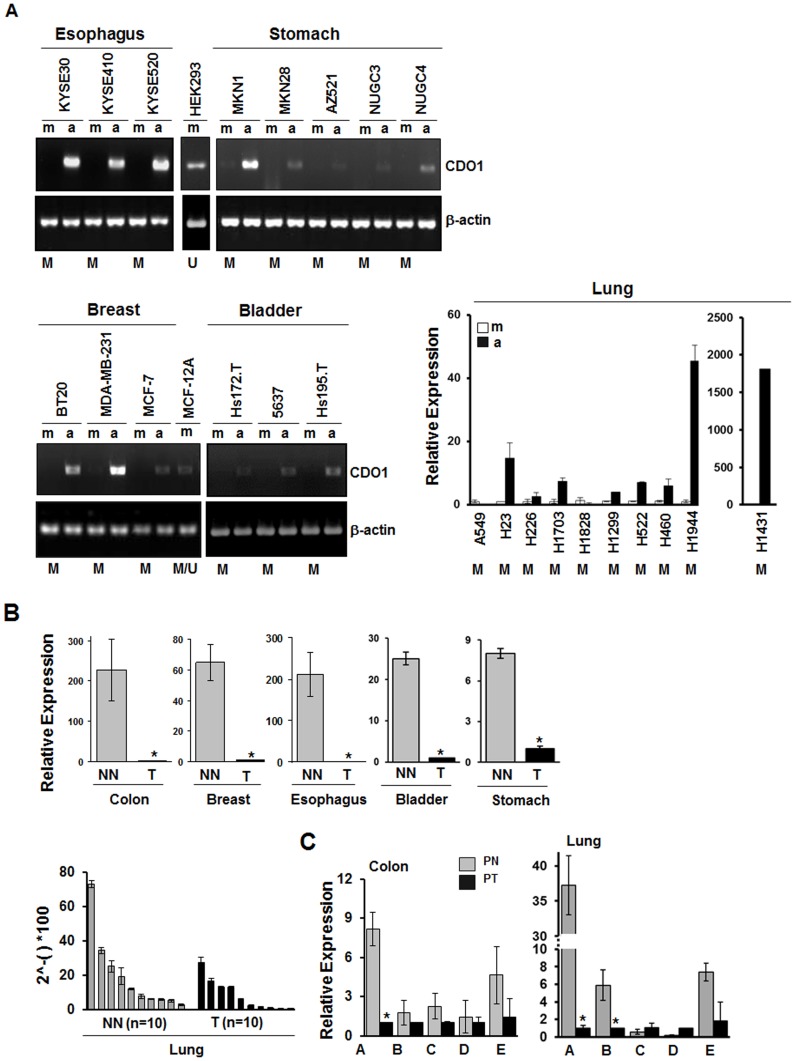
*CDO1* mRNA levels in different types of cancer. A , Expression of *CDO1* in cell lines was examined by RT-PCR or qRT-PCR analyses. m, mock treatment. a, 5-Aza-dC treatment (5 µM for three days). β-actin was used as a loading control. Methylation status of *CDO1* promoter in each cell line was examined and indicated as M for methylation, U for unmethylation, and M/U for co-existence of methylated and unmethylated alleles. *CDO1* was completely methylated in all cancer cell lines since only cytosine peaks were observed in CpGs sequenced (100% methylation) while it was not methylated in HEK293 since only thymidine peaks were observed (0% methylation). *CDO1* was partially methylated in MCF-12A since both methylated and unmethylated alleles were observed in 10 CpGs of the *CDO1* promoters examined. When a cytosine peak were compared with a thymidine peak in each CpG of the 10 CpGs, cytosine peaks were dominant (methylated), but since these “methylated CpGs” were found in less than 50% of total CpGs (10/34), it was considered as “methylation-negative” according to the criteria described in Materials and Methods. **B**. qRT-PCR was performed in cDNAs derived from patients with colon, breast, esophagus, bladder and stomach cancer (T) and patients without cancer (NN) (upper). Relative expression (Fold) was calculated by comparing the ratios of mRNA expression of *CDO1* to an internal control gene, β-actin. The *CDO1* expression level was determined in 10 lung cancer patients and 10 patients without cancer (lower). 2?-()*100, the expression of *CDO1* relative to β-actin calculated based on the threshold cycle (C_t_) as 2^−ΔCt^ (ΔCt = C_t,*CDO1*_ - C_t,β-actin_). Experiments were done in duplicate, and values indicate means ± SD. *, *P<0.05* in *T-*test. **C**, The *CDO1* expression level was examined in five pairs (A ∼ E) of matched cDNA prepared from patients with colon and lung cancer. PT, primary tumor; PN, matched normal tissues.

We then examined the transcriptional level of *CDO1* by RT-PCR and qRT-PCR analyses in cancer cell lines derived from breast, esophagus, lung, bladder, and stomach, and in non-tumorigenic cell lines (HEK293 and MCF-12A). Basal expression of *CDO1* was barely detectable in cancer cell lines but stronger expression was observed in HEK293 and weakly in MCF-12A cell lines (**Fig. 3A**). We also examined methylation status of the *CDO1* promoter in each cell line by bisulfite-sequencing, and found that cancer cell lines with *CDO1* loss harbored a dense methylation in the *CDO1* promoter (indicated as M). *CDO1* methylation was not observed in HEK293 cells (U), and both methylated and unmethylated alleles were found in MCF-12 cells (M/U). In addition, 5-Aza-dC treatment reactivated the *CDO1* expression in the majority of cancer cell lines (exceptions were A549 and H1828), indicating that transcriptional expression of *CDO1* tightly correlates with promoter methylation.

To examine the expression of *CDO1* in primary cancer, we performed qRT-PCR analysis with cDNAs derived from patients with colon, breast, esophagus, bladder and stomach cancer (T, n = 1 for each tumor) and patients without cancer (NN, n = 1 for each normal). *CDO1* was dramatically down-regulated in T compared to NN (**Fig. 3B** **, upper**). The overall mean value of the *CDO1* expression level in 10 patients without cancer was about 2.5 times higher (19.14±21.56) than that in 10 lung cancer patients (8.03±9.20) (**Fig. 3B** **, lower**). We also performed qRT-PCR in 5 pairs of matched cDNA prepared from tumor (PT) and corresponding normal tissues (PN) of colon and lung cancer. All five tumor cases of colon tissue exhibited *CDO1* down-regulation, and 3 of 5 cases of lung tissue displayed reduced levels of *CDO1* in PT compared to PN (**Fig. 3C**). These results suggest that specific decrease of *CDO1* mRNA is a common event in human cancer development.

**Figure 4:**
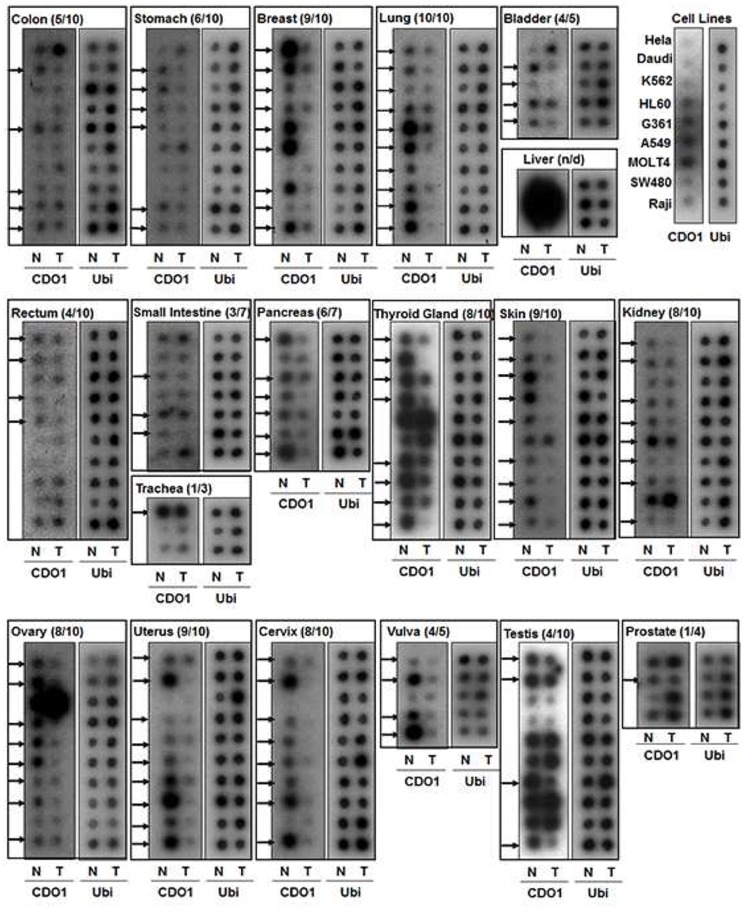
Investigation of *CDO1* expression with Cancer Profiling Arrays. Cancer profiling arrays II was performed to compare *CDO1* expression between tumor (T) and matched normal control (N) tissues of multiple tissue types. The array was hybridized with the *CDO1* cDNA probe labeled with ^32^P-α-deoxycytidine triphosphate according to the manufacturer’s protocol. Tissue type and the number of cases with down-regulation of *CDO1 vs.* total cases are indicated. Arrow, downregulation of *CDO1* in tumor compared to normal tissue. Ubiquitin cDNA (Ubi) was used as a control. n/d, not determined.

*CDO1* expression was also examined using Cancer Profiling Array II which includes normalized cDNA of tumor and the matched non-cancerous (normal) tissues for 19 different types of cancers. As demonstrated in **Figure 4**, *CDO1* expression was clearly detected in most corresponding normal tissues (N) in the array, and down-regulated in tumors in more than 50% of patients with colon, stomach, pancreas, thyroid, skin, kidney, bladder, lung, breast, ovary, uterus, cervix and vulva cancer. Conversely, an increase of *CDO1* in tumors was observed in less than 20% of patients with these types of cancer (**Table 2**). In lung cancer, all tumors (100%, 10 of 10) displayed down-regulation of *CDO1* (**Fig. 4** **, upper**), while the expression of *CDO1* in liver was too high to compare between normal and tumor tissues (not determined, n/d). In addition, a high frequency of *CDO1* down-regulation (>80%) was observed mostly in samples derived from female cancer patients (breast, ovary, uterus, cervix and vulva) (**Fig. 4** **, lower**). Thus, *CDO1* is downregulated in multiple human cancers, particularly in cancers from female organs.

**Table 2:** Expression of CDO1 in multple types of human cancer.

aTissue type	Down	Up	bn/c	Total cases
Colon	5	1	4	10
Stomach	6	1	3	10
Rectum	4	0	6	10
Small Intestine	3	1	3	7
Pancreas	6	0	1	7
Liver	cn/d	cn/d		3
Thyroid Gland	8	2	0	10
Skin	9	1	0	10
Kidney	8	0	2	10
Bladder	4	1	0	5
Trachea	1	0	2	3
Lung	10	0	0	10
Breast	9	0	1	10
Ovary	8	0	2	10
Uterus	9	0	1	10
Cervix	8	0	2	10
Vulva	4	0	1	5
Testis	4	2	4	10
Prostate	1	1	2	4

a Cancer profiling II was performed to compare CDO1 expression between tumor and corresponding normal cases of multiple tissue types.

b n/c, no change;

c n/d, not determined.

Note, CDO1 expression in normal and tumor liver could not be compared due to abundant expression of CDO1 in the tissue.

**Figure 5:**
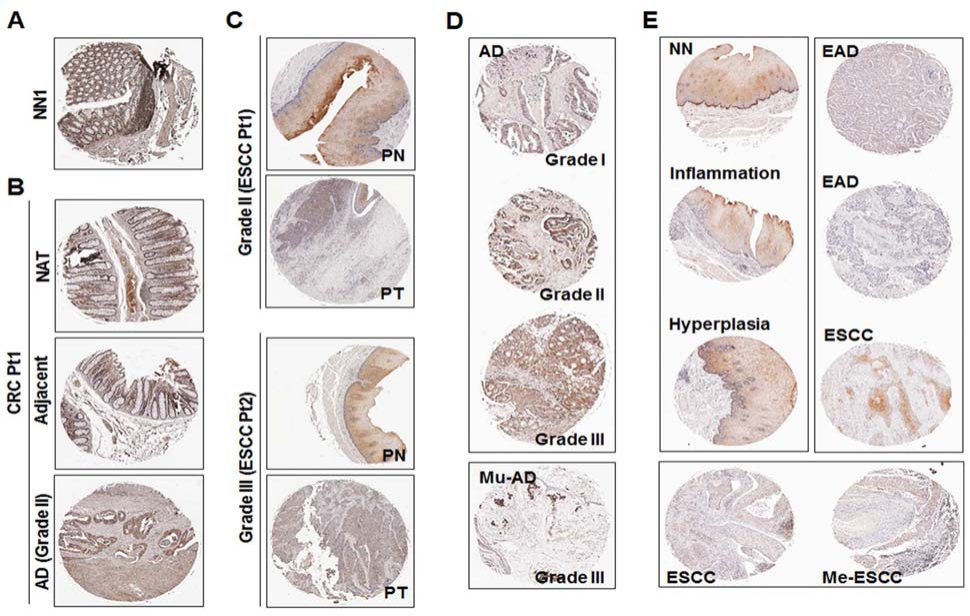
Immunohistochemical analysis of *CDO1* in colon and esophagus cancer tissue array. A , Strong expression of *CDO1* in non-malignant colon tissues. The expression of *CDO1* protein was positive in 6 out of 6 patients. NN1, a patient without cancer, and two more cases are shown in **Figure S2A**. **B**, A group of samples were derived from a single patient consist of colon adenocarcinomas (AD), matched cancer adjacent normal appearing tissue (NAT) and matched cancer adjacent tissues (Adjacent). *CDO1* expression was investigated in a total of 36 groups of samples. Patients were numbered arbitrarily (Pt1 ∼ Pt3). CRC Pt1, a patient with CRC. Two more cases are shown in **Figure S2B**. **C**, *CDO1* expression in ESCC. PT, ESCC; PN, matched normal appearing tissues. ESCC Pt1, a patient with ESCC. Three more cases are shown in **Figure S2C**. **D**, *CDO1* expression in colon adenocarcinoma (AD) with different tumor grades. Mu-AD, mucinous adenocarcinomas. **E**, *CDO1* expression in ESCC with different tumor grades. EAD, esophageal adenocarcinomas; Me-ESCC, metastatic ESCC.

To investigate protein expression of *CDO1*, we performed immunohistochemical (IHC) staining using both colon and esophagus tissue arrays with normal and cancer tissues. In the multiple organ normal tissue array that includes non-malignant tissues (NN) derived from esophagus, stomach, liver, colon, lung and breast (#BN00011), the expression of CDO1 protein was observed in all non-malignant tissues; CDO1 expression was positive in 6 out of 6 cases for all tissue types with only one exception in breast (4 out of 6 cases) (data not shown). **Figure 5A** and **Figure S2A** show representative results of three positive cases of non-malignant colon (NN1 ∼ NN3). In addition, among 36 groups of samples consisting of adenocarcinomas (AD), matched cancer adjacent normal appearing tissue (NAT) and matched cancer adjacent tissues (Adjacent) in each group that were derived from 36 individual colon cancer patients (#BC05021 and #BC05022), down-regulation of CDO1 was observed in 25 cases of AD compared to NAT or Adjacent tissue (69%) (**Fig. 5B** **& Fig. S2B**). Five cases of 36 AD displayed up-regulation of CDO1, but the other six cases exhibited no difference among AD, NAT and Adjacent tissues (data not shown). The high frequency of decreased CDO1 expression was also observed in 33 out of 40 cases of ESCC (PT) compared to matched normal appearing tissues (PN) (82%) (**Fig. 5C** **& Figure S2C**). Only two cases of ESCC exhibited increased CDO1 expression, and five cases showed no difference between PT and PN (data not shown) (#ES801). CDO1 expression was then analyzed in colon adenocarcinoma with different tumor grades and NAT (#BC05118 and #CO1922). CDO1 protein was detected in 41 out of 41 cases (100%) of NAT, whereas AD displaying CDO1 positivity was about 40% (37% & 44%, respectively) (**Fig. 5D** **&** **Table 3**). Faint or absent expression of CDO1 in AD was statistical significant when compared to NAT in both arrays (*P<0.001*), and the absent expression of CDO1 in mucinous AD (84%, 32/38) was also statistically significant (*P<0.001*). CDO1 expression was detected in 30 out of 30 cases (100%) of non-malignant esophageal tissues (normal, inflammation and hyperplasia), whereas absent or faint expression of CDO1 was observed in 35 out of 49 cases (71%) of neoplastic tissues (**Table 4**) (#ES804). When compared to normal esophagus tissue, the negative expression of CDO1 in esophageal AD (80%, 16/20) (*P<0.001*), SCC (55%, 9/20) (*P<0.001*), and metastatic cancer (89%, 8/9) (*P<0.001*) was statistically significant but this was not the case in hyperplasia (100%, 10/10) (**Fig. 5E**).

### *CDO1* Controls Tumorigenicity of Human Cancer Cells *in vitro* and *in vivo*

To gain insight into the function of *CDO1* and the consequence of a loss of *CDO1* activity, we first examined the growth properties of cancer cell lines with or without forced expression of *CDO1*. HCT116 and DLD-1 cell lines, which do not express *CDO1* gene at baseline, were selected for transient *CDO1* gene delivery. We performed MTT assay to compare cell growth with or without expression of *CDO1*. The control cells which did not express *CDO1* consistently grew for 3 days of incubation, but the growth in *CDO1* expressing HCT116 and DLD-1 cells decreased to 42% and 27% of the control cells, respectively (**Fig. S3A**). However, ectopic expression of *CDO1* had no effect on cell morphology (data not shown). We then performed a colony focus assay after incubating cells in the presence of G418 for 2 weeks. In control cells, both cell lines exhibited strong colony-forming ability with multiple colonies (200±45.3 colonies for HCT116 and 300±35.3 colonies for DLD-1) (**Fig. S3B**). However, in p*CDO1*-transfected cells, a marked decrease in colony numbers was observed (20±12.2 for HCT116 and 80±11.3 colonies for DLD-1) (*P*<0.001). Similar results were observed in p*CDO1*-transfeced KYSE30, MCF-7, NUGC3 and H1431 cells (**Fig. S3C**), indicating that forced expression of *CDO1* suppresses *in vitro* cell growth. To investigate whether CDO1 has apoptotic activity, we assessed the apoptotic cell population by flow cytometry after staining cells with Annexin V-FITC and 7-AAD. No apoptosis was observed in p*CDO1*-transfected HCT116 and DLD-1 cells compared to the control (data not shown), suggesting that reduced cell growth is due to a decreased proliferation rate not apoptosis.

**Figure 6:**
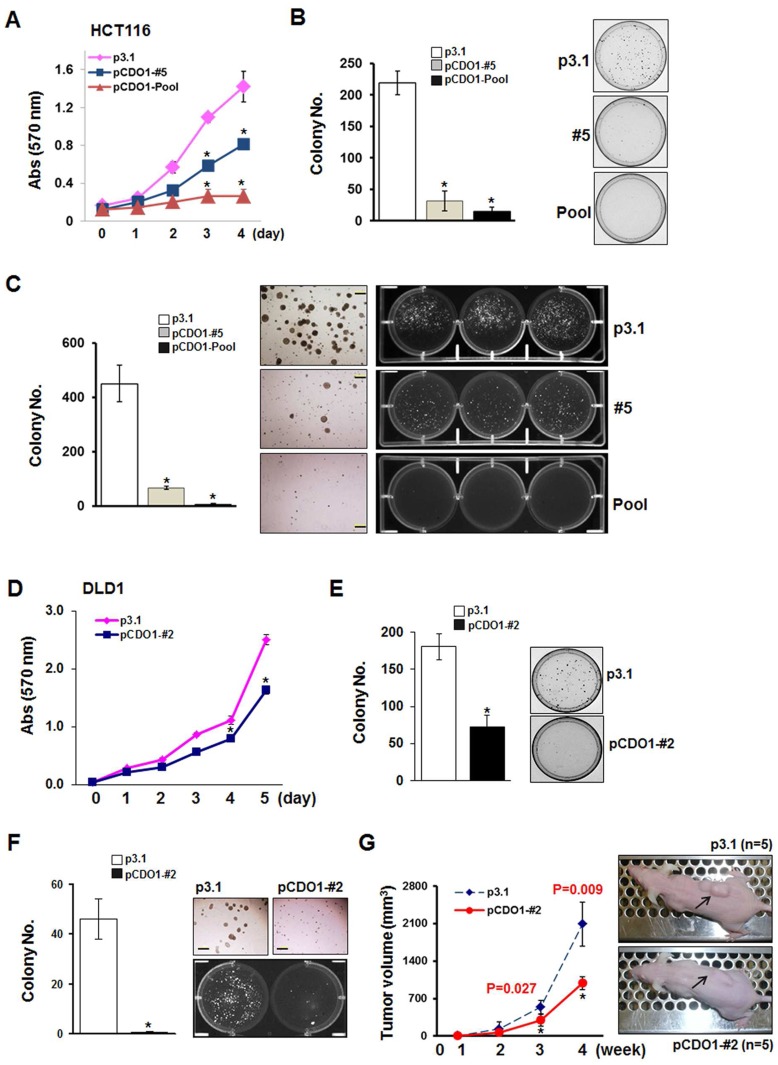
Tumor suppressive role of *CDO1*. A . The MTT assay was performed in HCT116 clones stably expressing *CDO1* or control clones. Cell growth was expressed as absorbance (Abs) at 570 nm wavelength. Two independent experiments were done in triplicate, and values are expressed as means ± SD. *, *P<0.05* in *T-*test. **B**, Colony focus assays were performed in the HCT116 clones after incubation in the presence of G418 for 10 days. Colonies were stained with 0.4% crystal violet solution (MeOH/10% Acetic acid, 3∶1). After air-drying, colonies were photographed under a microscope (right). Values are expressed as means ± SD and are derived from experiments done in triplicate. **C**, Inhibitory ability of *CDO1* in anchorage-independent cell growth was determined by the soft agar assay. Colonies at size >0.5 mm were counted (left). Colonies were photographed under phase-contrast microscope (middle) or under UV after staining with ethidium bromide in 1 × PBS/0.1% Triton X-100 solution overnight (right). Scale bar, 500 µm. **D**, The MTT, colony focus (**E**) and soft agar assays (**F**) were performed in DLD-1 clones stably expressing *CDO1* or control clones. **F**, Colonies were photographed under a phase-contrast microscope (right top) or under UV after staining with ethidium bromide in 1 X PBS/0.1% Triton X-100 solution overnight (right bottom). **G**, DLD-1-p*CDO1*-#2 and DLD-1-p3.1 cells were injected on the right flank of 6-week-old nude mice (n = 5 each), and the time course of tumor growth was measured once a week for 4 weeks with caliper (left). Each point represents the mean ± S.D. of tumor volumes of mice in each group. At day 28 after injection, pictures were taken before mice were sacrificed (right). *, *P<0.05* in *T-*test.

To further study the tumor suppressor activity of *CDO1* in CRC cell lines, we established clones stably expressing *CDO1* and then selected clones with relatively high levels of *CDO1* in HCT116 (p*CDO1*-#5 and p*CDO1*-Pool) and DLD-1 cells (p*CDO1*-#2) (**Fig. S3C**). We examined *in vitro* cell growth in the *CDO1* clones and in the control cells (vector alone, p3.1) by MTT assay. Compared to the control cells that grew exponentially, cell growth in the *CDO1* clones was delayed (**Fig. 6A & 6D**). We also confirmed a marked decrease of colony forming ability of the *CDO1* expressing cells compared to control (**Fig. 6B & 6E**). To determine if *CDO1* inhibited anchorage-independent cell growth, we assessed colony formation in soft agar. We found a remarkable decrease in the number of colonies formed by the *CDO1* clones compared to control (**Fig. 6C & 6F**). Thus, *CDO1* can suppress cell growth and anchorage independence of human colon cancer cells. In addition, we performed the *in vitro* cell invasion assay, and found that the number of cells passing into the invasion chamber decreased significantly in HCT116 cells stably expressing compared to the control. A significant inhibition was also found in the clone derived from DLD-1 (**Fig. S3D**). To examine the tumor suppressive role of *CDO1* in an *in vivo* mouse model, we injected subcutaneously DLD-1-p*CDO1*-#2 and DLD-1-p3.1 clones into the right flanks of 6-week-old nude mice. Tumor volume (mm^3^) was measured once a week for 4 weeks after injection, and tumor development of the control and CDO1 clones was first observed at day 8 and 11, respectively. A significant decrease of tumor volume was observed at the 4^th^ week in mice injected with CDO1-expressing cells (989±408.1 mm^3^) compared to control mice (2,091±119.2 mm^3^) (*P = 0.009*) (**Fig. 6G**). Taken together, these results indicate that CDO1 suppresses the *in vitro* and *in vivo* tumorigenicity of human colon cancer cells.

**Table 3:** Immunohistochemical analysis of CDO1 in colon cancer tissue microarray.

Tissue Array		Grade	*CDO1* (+)	%	*P value*
**BC05118**	a **Normal**	**Total**	**41/41**	**100**	
	b **Adenocarcinoma**	**Total**	**28/48**	**58.3**	<0.001*
		* I*	9/19	47.4	
		* II*	16/22	72.7	
		* III*	3/7	42.8	
**CO1922**	c **Adenocarcinoma**	**Total**	**116/182**	**63.7**	<0.001*
		* I*	21/33	63.6	
		* II*	74/110	67.3	
		* II-III*	6/9	66.7	
		* III*	15/30	50.0	
	d **Mucinous Adenocarcinoma**	**Total**	**6/38**	**15.8**	<0.001*

Expression level was indicated as -, absent or faint expression; +, moderate expression; ++, expression; +++, strong expression.

CDO1 positivity (+) was counted in samples with over moderate expression.

P values from Fisher’s exact test performed in Normal vs. adenocarcinoma in BC05118, adenocarcinoma in CO1922,

or mucinous adenocarcinoma in CO1922. *P<0.05 considered significant.

a Cancer adjacent normal colonic tissue that excluded 5 cases of smooth muscle and 4 cases of disrupted tissue (not determined).

b A case (fibrous tissue) was excluded.

c Ten cases of adenocarcinoma without grade and two cases squamous cell carcinoma were excluded,

but 38 cases of mucinous adenocarcinomas were included.

d Tumor grade was not considered.

**Table 4:** Immunohistochemical analysis of CDO1 in esophageal cancer tissue microarray with normal tissue controls (ES804).

		Grade	*CDO1* (+)	%	*P value*
a **Normal**	**Total**		**30/30**	**100**	
	Normal		10/10	100	
	Chronic esophagitis		10/10	100	
	Hyperplasia		10/10	100	
**Malignant**	**Total**		**14/49**	**28.6**	
	Adenocarcinoma	Subtotal	4/20	20.0	*<0.001* *
		* II*	2/10	20.0	
		* II–III, III*	2/8	25.0	
		* Adenosquamous carcinoma*	0/1	0	
		* Basaloid squamous cell carcinoma*	0/1	0	
	Squamous cell carcinoma	Subtotal	9/20	45.0	*<0.001* *
		* I*	7/11	63.6	
		* I–II, II*	1/5	20.0	
		* III*	1/4	25.0	
	Metastatic cancer	Subtotal	1/9	11.1	*<0.001* *
		Metastatic adenocarcinoma	0/3	0	
		Metastatic squamous cell carcinoma	1/6	16.7	

Expression level was indicated as −, absent or faint expression; +, moderate expression; ++, expression; +++, strong expression.

CDO1 positivity (+) was counted in samples with over moderate expression.

P values from Fisher’s exact test performed in normal vs. adenocarcinoma, squamous cell carcinoma, or metastatic cancer.

* P<0.05 considered significant.

a Non-malignant normal esophagus tissue and cancer adjacent normal esophagus tissue.

### Knockdown of *CDO1* Increases *in vitro* Cell Growth

**Figure 7:**
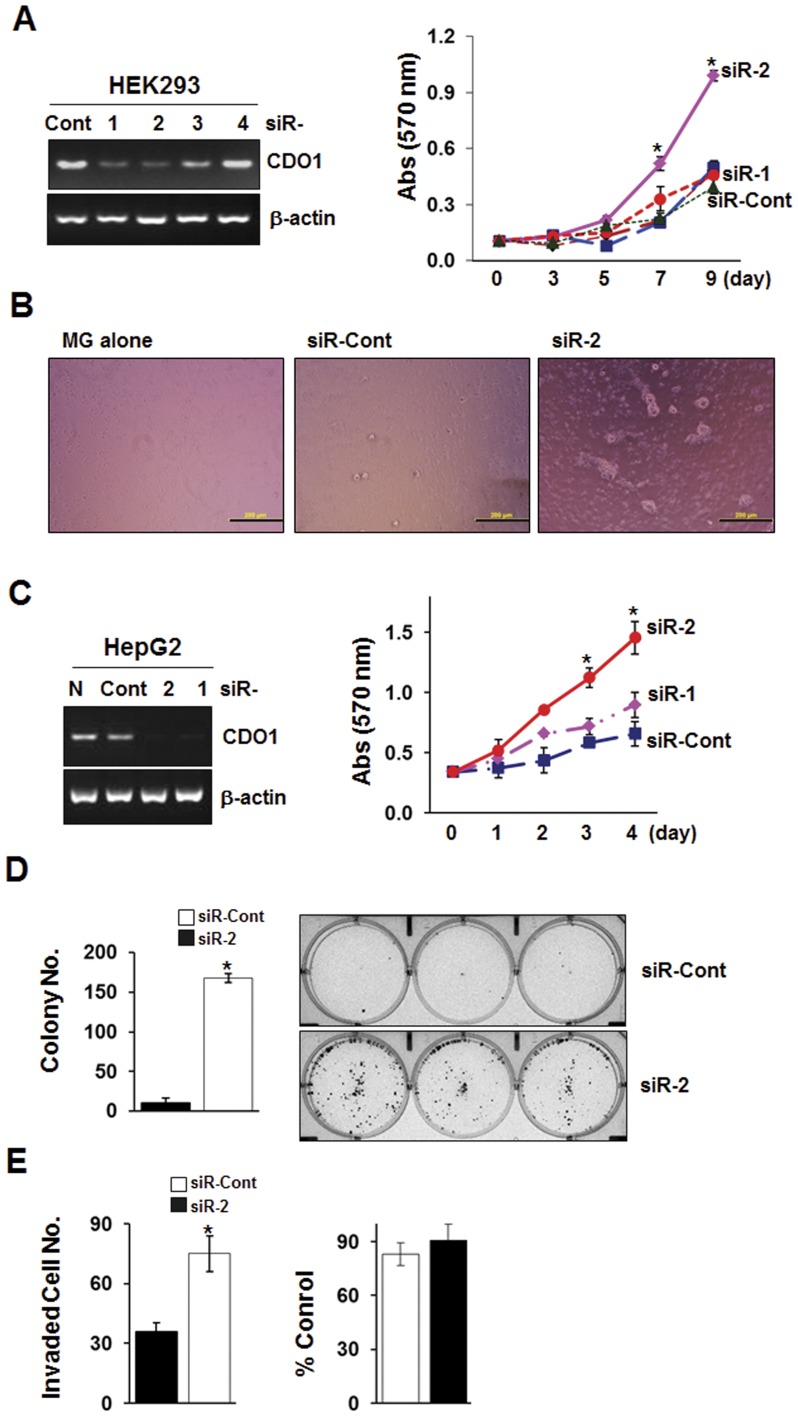
Increased cell growth by *CDO1* knockdown. **A**, siRNAs targeting *CDO1* mRNA (siR-1 ∼ -4) and a non-targeting control siRNA (siR-Cont) were transfected into *CDO1*-expressing HEK293 cells, and *CDO1* gene knockdown was examined by RT-PCR analysis (left) and cell growth was determined by the MTT assay (right). β-actin was used as a loading control. Two independent experiments were done in triplicate, and values are expressed as means ± SD. *, *P<0.05* in *T-*test. Both siR-1 and 2 reduced the *CDO1* mRNA, but only siR-2 displayed increased HEK293 cell growth (left). **B**, The morphology of HEK293 cells were examined under a phase-contrast microscope after siRNA-transfected cells were grown on Matrigel beds for 3 days. MG alone, a picture of Matrigel without cells. Scale bar, 500 µm. **C**, HepG2 cells express *CDO1*, but do not harbor the gene methylation (data not shown). *CDO1* siRNA (−1 and −2) or control siRNA were transfected into HepG2 cells, and RT-PCR and MTT assays were performed. N, cells without transfection. Increased cell growth was observed in cells transfected only with siR-2. **D**, Colony focus assays were performed in HepG2 cells after transfection with siR-2 and controls. Colonies were grown for 13 days and stained with crystal violet solution. After air-drying, colonies were counted (left) and photographed (right). Two independent experiments were done in triplicate, and values are expressed as means ± SD. *, *P<0.05* in *T-*test. **E**, The *in vitro* cell invasion assay was performed in the HepG2 cells after transfection with siR-2 and controls. Cells were incubated for 16 hrs, and after fixation and staining, invading cells were counted at 100 X magnification (left). Cell growth for 16 hrs determined by MTT assay was not significant (right).

To demonstrate the effects of knockdown of *CDO1*, we transiently transfected siRNAs targeting *CDO1* mRNA (siR-1 ∼ −4) and a non-targeting control siRNA (siR-Cont) into CDO1-expressing HEK293 and HepG2 cell line, and determined *CDO1* gene knockdown by RT-PCR. As shown in **Figure 7A and 7C** (**left**), among 4 individual *CDO1* siRNAs, siRNA-1 and -2 were effective in reducing *CDO1* mRNA level in the HEK293 and HepG2 cells. We then performed the MTT and colony focus assays to determine the cell growth. Reduced CDO1 expression resulted in increased cell growth of both HEK293 and HepG2 cells (**Fig. 7A & 7C** **, right**). We examined morphologic differences of HEK293 cells after growing them on Matrigel beds for 3 days. Control cells formed colonies only poorly on the Matrigel, whereas cells transfected with *CDO1* siR-2 grew in clumps and piled up, and increased colony numbers compared to the control (**Fig. 7B**). In addition, a significant increase of clonogenic cell growth (16-fold) was observed in HepG2 cells transfected with siRNA-2 whereas much smaller colonies from the control cells were observed in 13 days of incubation (**Fig. 7D**). Increase of the *in vitro* invasive activity (2-fold) was also observed in HepG2 cells with diminished *CDO1* expression (**Fig. 7E**). These data indicate that cells with loss of *CDO1* can increase cell growth and invasion capacity.

## Discussion

In this study, we describe the identification of *CDO1* promoter methylation, a specific marker for multiple types of human cancer. We found a statistically significant difference in the frequency of *CDO1* promoter methylation between primary normal and tumor tissues derived from colon, breast, esophagus, lung, bladder and stomach. The mRNA expression of *CDO1* was silenced in all cancer cell lines tested but re-activated by the demethylating agent, 5-Aza-dC. *CDO1* was also down-regulated in primary tumors derived from colon, breast, esophagus, lung, bladder, and stomach compared to normal tissues. In addition, *CDO1* displayed tumor suppressive activities in an *in vitro* cell culture and an *in vivo* mouse model. These results suggest that *CDO1* is a novel tumor suppressor epigenetically regulated in human cancer.

A mutation in the *CDO1* gene (115170091G>C) has been found in a xenograft of metastatic CRC (liver metastasis, cancer stage IV) [29]. However, we could not find the mutation in all 5 CRC cell lines and 100 primary colon tissues in our study (data not shown), suggesting that promoter methylation may play a major role in inactivation of *CDO1* gene in CRC. Promoter methylation of *CDO1* has been described in a few reports so far. Using a genome wide gene expression microarray, Maschietto et al. [30] observed *CDO1* to be downregulated in relapsed Wilm’s tumor, a pediatric kidney tumor. Their group did not observe re-expression of this gene after demethylating agent treatment in cell lines, and they did not examine its promoter for methylation status. Combining genome wide expression analysis and a genome wide methylation analysis, Kwon et al. [31] identified *CDO1* to be downregulated and hypermethylated in lung SCC. They observed re-expression of the *CDO1* after 5-Aza-dC treatment in lung cancer cell lines. *CDO1* promoter methylation was also reported to be a strong predictor for distant metastasis in cohorts of lymph node-positive/ER-positive breast cancer patients who underwent anthracycline-based chemotherapy [32]. It is known that 5-Aza-dC treatment reactivated the *CDO1* gene transcript in CRC cell lines (HCT116 and SW480) by inducing a localized hypomethylation in the promoter proximal to TSS [33], which is consistent with our results. These results further support our hypothesis that cancer-specific methylation and decrease of *CDO1* expression may be common events in human cancer development.

Histone modifications are surely one of the important methods involved in gene repression regulation. Mossman and Scott [33] demonstrated in 2 colon cancer cell lines (HCT116 and SW480), treatment with 5-Aza-dC was able to induce gene expression of *CDO1*, but this expression was not complete and could be reverted after a few days. They used chromatin immune-precipitation to test if chromatin modifications were also playing a role in gene expression, together with DNA methylation. Their results suggest that demethylation induced by 5-Aza-dC and increased H3K4me3 allow initiation of expression and H3K27me3 seems to further increase transcription. This notion corroborates the idea of distinct epigenetic mechanisms playing together in gene expression regulation. It suggests that further studies should include chromatin modifications analysis to complement DNA methylation analysis of *CDO1* in patients’ samples, once this was only tested in cell lines.

Cysteine homeostasis is very important to the living organism; it is dependent on the regulation of CDO that oxidizes cysteine to cysteine sulfinate, which is further metabolized to either taurine or to pyruvate plus sulfate [34], [35]. When cysteine supply is abundant due to an increase of sulfur amino acid intake, cells increases rapidly the catalytic activity of the CDO to prevent cysteine cytotoxicity. Thus, cysteine levels in the body are controlled predominantly via regulation of CDO concentration and activity [35], [36].

The importance of CDO in human health is suggested by evidence for abnormal or deficient CDO activity in individuals with several autoimmune (e.g., rheumatoid arthritis) and neurodegenerative diseases (e.g., Parkinson’s and Alzheimer’s disease) ([16], [17], [37], [38], [39]). The etiology of these diseases is linked to functional impairment of CDO, which might lead to elevated levels of cysteine and H_2_S as well as lowered concentrations of taurine and sulfate. Although we did not measure the levels of these metabolites in our tumor and normal tissue samples, a few reports suggest an increase of cysteine level in human cancer; Ishimoto et al. reported that CD44, an adhesion molecule expressed in cancer stem-like cells, regulates redox status and promotes tumor growth [40]. In the report, they showed that siRNA-mediated CD44 gene knockdown decreased the level of cysteine in HCT116 cells [40]. Interestingly, cysteine level increased in tissues of metastatic prostate cancer (Mets) and clinically localized prostate cancer (PCA) compared to benign adjacent prostate (Benign), whereas taurine level decreased in PCA and Mets compared to Benign [41]. These results implicate that a high level of cysteine alone or the cysteine-to-taurine ratio can be a biomarker in human cancer.

Ueki et al. have recently reported that *cdo* gene knockout (*cdo* ^−/−^) mice exhibited postnatal mortality, growth deficit, and connective tissue pathology, and had extremely low taurine levels, consistent with the lack of flux through CDO-dependent catabolic pathways [42]. Interestingly, despite the block in CDO-dependent catabolism of cysteine, *cdo* ^−/−^ mice exhibited mildly elevated levels of cysteine, and higher levels of sulfate than *cdo* ^+/+^ mice. These results indicate that *cdo* ^−/−^ mice increased catabolism of cysteine by the CDO-independent desulfuration pathways which release reduced sulfur (H_2_S, or HS^−^) and its oxidized form, sulfate [43], [44], [45]. If an increase of cysteine level is a common event in human cancer, tumors need mechanisms to avoid cysteine toxicity resulting from disturbance of cysteine homeostasis by tumors themselves. Tumors may use the CDO-independent desulfuration pathways or unknown mechanisms to increase of catabolism of cysteine or to maintain cysteine level below the cytotoxic concentration. The mechanisms to render tumor cells resistant to high levels of cysteine during human carcinogenesis needs to be further investigated.

Lack of CDO activity is partially responsible for sulfide (H_2_S, or HS^−^) production from cysteine. H_2_S inhibits cytochrome c oxidase (COX), the terminal oxidase of the mitochondrial electron transport chain [46]. However, sulfide is not a simple toxic molecule; it is believed to be an important gaseous signaling molecule in eukaryotes [47], [48], [49], [50]; it causes smooth muscle relaxation, thereby regulating vascular tone, intestinal contractility, and myocardial contractility [51], [52], [53], [54], [55]. In the central nervous system, H_2_S enhances synaptic transmission by increase of the sensitivity of *N*-methyl-D-aspartate receptors to glutamate in hippocampal neurons [56], [57], [58]. H_2_S may activate anti-inflammatory and anti-oxidant pathways [59], [60], and have protective effects against oxidative stress [61], [62], ischemia-reperfusion injury [63], [64], [65], and certain toxins [66], [67]. In Parkinson’s disease models, H_2_S inhibits rotenone-induced apoptosis via preservation of mitochondrial function [68]. In addition, H_2_S is present in the lumen of the human large intestine at millimolar concentrations, and micromolar concentration of H_2_S increases respiration of colonic epithelial cells and energizes mitochondria allowing these cells to detoxify and to recover energy from luminal sulfide [69].

Dysregulation of H_2_S production is connected to connective tissue disorder in individuals with impaired collagen and elastin synthesis (e.g., Ehlers-Danlos syndrome) and patients with a deficiency of mitochondrial sulfur dioxygenase (ETHE1) [70]. COX activity is reduced in muscle, brain, and colon of individuals with ethylmalonic encephalopathy which is caused by mutations in the ETHE1, and in muscle, brain, and liver of *Ethe1* ^−/−^ mice [71], [72]. In the study of Ueki et al., *cdo* ^−/−^ mice exhibited a higher level of acid-labile sulfide in tissue, and loss of mitochondrial COX activity than *cdo* ^+/+^ mice [42], indicating that H_2_S may mediate phenotypic changes common in both *cdo* ^−/−^ and *Ethe1* ^−/−^ gene disruptions. In contrast, blood glucose level decreased in *cdo* ^−/−^ mice [42], and the level of lactic and pyruvic acid increased in *Ethe1* ^−/−^ mice [22020834], suggesting an increased anaerobic glycolysis in both *cdo* ^−/−^ and *Ethe1* ^−/−^ mice.

Imbalance between the luminal concentration of free sulfide and the capacity of colonic epithelial cells to metabolize H_2_S may impair O_2_ consumption in the colonic epithelial cells, leading to mucosal insult, inflammation, and ultimately in colorectal cancer (CRC) [69]. It is known that exogenously administered H_2_S at micromolar concentration also induced human colon cancer cell proliferation by increase of Akt and ERK phosphorylation [73]. At millimolar concentration, NaHS increased the proliferation in nontransformed rat intestinal epithelial cells, but inhibited mitochondrial respiratory activity [74]. In addition, dysregulation of thiosulfate sulfurtransferase (TST) located on the luminal mucosal surface of normal colon results in ineffective detoxification of H_2_S, and expression of TST is lost in ulcerative colitis and advanced colon cancer, and reactivated by histone deactylase inhibitors [75], indicating that TST may be a factor for the cell loss and inflammation that accompany ulcerative colitis and ultimately CRC. These results suggest that sulfide may initiate epithelial cell dysregulation, which may contribute to CRC development.

A strong sexual dimorphism of cysteine metabolism in male and female mice has been reported [42]; in wild-type mice, CDO and taurine levels were higher in female than male mice, suggesting that more cysteine is catabolized in female mice. When the *cdo* gene was disrupted, female mice displayed a higher incidence of postnatal mortality than did male mice, and taurine supplementation improved weight gain and lowered cysteine levels in female *cdo* ^−/−^ offspring, but not in male *cdo* ^−/−^ offspring. In our results, a high frequency of *CDO1* down-regulation (>80%) was observed in female cancer patients, but not in male patients (<40%). Whether women are more sensitive to cysteine catabolism or vulnerable to the *CDO1* loss-mediated carcinogenesis than men will be of interest in future study.

Although there is no evidence that the concentration of endogenously produced H_2_S increases in human cancer, it is possible that tumors with loss of the *CDO1* gene may have capacity to adapt to an excess in luminal sulfide production, capacities to detoxicate and to use sulfide as an energy source, or availability of anaerobic metabolic pathway (i.e., glycolysis) for energy production when mitochondrial oxygen consumption is impaired (Warburg effect) [69]. Increased aerobic glycolysis and mitochondrial dysfunction are common features of aggressive cancer growth [76]. If epigenetic silencing of the *CDO1* gene is responsible for enhanced aerobic glycolysis and mitochondrial dysfunction in human cancer, H_2_S may be a key molecule to activate the glycolytic pathway and accelerate mitochondrial dysfunction. As observed in *cdo* ^−/−^ mice, *CDO1* gene loss in human cancer increases the accumulation of cellular cysteine, which is further catabolized to acid-labile sulfide. Tumors may use the sulfide as a survival signal for preserving mitochondrial function or for enhancing aerobic glycolysis. Therefore, of potential interest for future studies will be the role of CDO1 in mitochondria and glycolysis and the signaling mechanism of H_2_S as well as the level of sulfide in tumors.

In conclusion, our data implicates cancer-specific methylation and silencing of *CDO1* as a common inactivation event in human carcinogenesis. Detection and quantification of *CDO1* methylation deserves further attention as a diagnostic biomarker of major human cancers due to its high frequency in primary tumors and near absence in normal tissues. Detection of *CDO1* methylation levels in stool and/or plasma DNA may prove value of the gene in the diagnosis and monitoring of cancer patients. In addition, our results provide tantalizing clues for new approaches in the treatment of human cancer. Further evaluation of the effect of H_2_S on the mechanisms of growth regulation may provide new therapeutic approaches for the treatment of human cancer patients.

## Materials and Methods

### Cell Lines

We used 6 different human cancer cell lines in this study. All of the cell lines in this study were purchased from ATCC (Manassas, VA, USA) or other sources were previously described [25], [26], [77], [78], [79]; CRC cell lines (HCT116, HT29, DLD-1, RKO and SW480), ESCC cell lines (KYSE30, KYSE410 and KYSE520), gastric cancer cell lines (MKN1, MKN28, AG521, NUGC3 and NUGC), breast cancer cell lines (BT20, MDA-MB-231, MCF-7, Hs748.T, Hs578.T), bladder cancer cell lines (Hs172.T, 5637 and Hs195.T), lung cancer cell lines (A549, H23, H226, H1703, H1828, H1299, H522, H460, H1944 and H1431), HepG2 human hepatocellular carcinoma cell line, and non-tumorigenic cell lines (MCF-12A breast epithelial cell line and HEK293 human embryonic kidney cell line). Cell lines were propagated in accordance with the instructions from American Type Culture Collection.

### Tissue Samples

Sample cohorts used in this study were previously described [22]. Tissue samples from 6 different types of primary cancers (CRC, ESCC, stomach, breast, bladder and lung) were microdissected to isolate >70% epithelial cells in both neoplastic and nonneoplastic tissues. Tissue samples from age-matched individuals without a history of malignancy (NN) were used as controls, and gDNA from primary cancers (PT) and matched normal adjacent tissue (PN) were described previously [22]. Written informed consent was obtained from the patients who provided the tissues, and this study was approved by the Institutional Review Board of the Johns Hopkins University (IRB 03–11–12-06e). This study qualified for exemption under the US Department of Health and Human Services policy for protection of human subjects (45 CFR 46.101(b)).

### mRNA Expression Microarray

We performed oligonucleotide microarray analysis on the GeneChip Human Genome U133A Array (Affymetrix) containing 22,284 genes as per the manufacturer’s instruction, and details were previously reported [26]. We identified genes absent at baseline or upregulated by pharmacological treatment according to the manufacturer’s algorithm.

### Sequencing and Combined Bisulfite Restriction Analysis (COBRA)

All PCR reactions were done as described previously [26], and the primer sequences of bisulfite-DNA amplification were described previously [26]. PCR products were gel-extracted (Qiagen, Valencia, CA) and sequenced with forward primer (F1) using the ABI BigDye cycle sequencing kit (Applied Biosystems, Foster City, CA). Searches for CpG islands in each gene promoter were done by using the online accessible software Methprimer. Bisulfite-sequencing primers were designed at the CpG islands within 1 kb upstream of the transcription start site (TSS). For COBRA, eluted DNA after gel extraction was digested with *BstU1* (New England Biolabs., Beverly, MA), which recognizes the CGCG sequence, for 3 hrs at 60°C. Samples were loaded on a 10% acrylamide gel, stained with 1X SYBR Green Gold (Molecular Probes, Eugene, Oregon), and visualized under UV light.

### The Criteria to Determine Methylation in Cell Lines and Tissues

Bisulfite-sequencing was based on nucleotide sequences in electropherograms. When only a cytosine or a thymidine peak existed in a CpG, the sequence was “CG” (100% methylation) or “TG” (0% methylation). When both methylated and unmethylated alleles were observed in a CpG sequence, it was considered as “partially methylated” (M/U). When “partial methylated CpG” was observed, a cytosine peak was compared to a thymidine peak in the CpG. If a cytosine peak was similar to a thymidine peak or dominant, the sequence in electropherograms was “NG” or “CG”, indicating that over 50% methylated alleles existed. When a thymidine peak was dominant, the sequence was “TG”, indicating less than 50% methylated alleles. Only “NG” and “CG” were considered as “methylated” in the CpG. When “methylated” CpG was found in more than 50% of total CpGs in an amplified PCR product, it was considered as “methylation-positive.” When a sample was “methylation-positive,” it was classified as “methylation (M)”.

### Quantitative Methylation-specific PCR (TaqMan-MSP)

For quantitative methylation analysis, PCR primers were designed to hybridize to the region of *CDO1* gene that was determined to be methylated in CRC cell lines by bisulfite-sequencing, and a fluorescent probe was synthesized to the amplified region of the DNA. Primer and probe sequences for TaqMan-MSP are shown in **Table S1**. All oligonucleotide primer pairs were purchased from Invitrogen (Carlsbad, CA), and the TaqMan probe from VWR (West Chester, PA). All protocols for TaqMan-MSP were performed as reported [23], and all reactions were performed in duplicate. The methylation ratio (TaqMan methylation value, TaqMeth V) was defined as the quantity of fluorescence intensity derived from promoter amplification of *CDO1* gene divided by fluorescence intensity from β-actin amplification, and multiplied by 100. This ratio was used as a measure for the relative level of methylated DNA in samples.

### 5-Aza-dC Treatment, RT-PCR and the Quantitative Real-time RT-PCR

Cells were treated with 5 µM 5-aza-2′-deoxycytidine (5-Aza-dC) (Sigma, St. Louis, MO) every 24 hrs for 3 days. RNA was extracted using Trizol (Invitrogen, Carlsbad, CA) and reverse-transcribed with Superscript II reverse transcriptase (Invitrogen). RT-PCR was performed by 30 cycles of 95°C for 1 min, 58°C for 1 min, and 72°C for 1 min. PCR products were gel-extracted and sequenced to verify true expression of the genes. For the qRT-PCR analysis, five matched normal and tumor cDNA (A ∼ E) were purchased from Clontech Laboratories, Inc. (Mountain View, CA). cDNA panels of human normal (NN) and cancer tissue (T) derived from colon, breast, esophagus, bladder and stomach were purchased from BioChain Institute, Inc. (Hayward, CA). One µl of each cDNA was used for qRT-PCR using QuantiFast SYBR Green PCR Kit (Promega, Valencia, CA) as described [23]. Expression of genes relative to β-actin was calculated based on the threshold cycle (C_t_) as 2^–Δ(ΔCt)^, where ΔCt = C_t,*CDO1*_ −C_t,*β-actin*_ and Δ(ΔCt) = ΔCt,_N_ −ΔCt,_T_ (N, normal tissue cDNA; T, tumor tissue cDNA). Primer sequences are shown in **Table S1**.

### Construction of Luciferase Vectors and Reporter Assay

Potential *CDO1* promoter regions upstream of the transcription start site (TSS) (−1100 and −430 bp to +104 bp) were prepared by PCR using pfx DNA polymerase (Invitrogen). Genomic DNA extracted from the HCT116 human colon cancer cell line was used as template. The forward primers were synthesized corresponding to the upstream sequences of desired promoter regions and the reverse primers included bp +104 relative to the reported TSS of the human *CDO1* gene. 5′- flanking *KpnI* and A 3′- flanking *XhoI* site were added to forward and reverse primers, respectively. The pGL2 promoter control vector (Promega, Madison, WI) was digested with both *KpnI* and *XhoI* and treated with calf intestinal alkaline phosphatase. The PCR fragments were digested with *KpnI* and *XhoI*, and ligated with phosphatase-treated pGL2 vector to generate pGL2-*CDO1*-Luciferase constructs (#1 and #2). Plasmids were transfected into HEK293 and HCT116 at a density of 1×10^5^/well in a 24-well plate. For each well, 100 ng of the pGL2-*CDO1*-Luciferase constructs was co-transfected with 10 ng of internal control reporter pSV-*Renilla* (Promega) using Fugene-6 (Roche, Basel, Switzerland) in accordance with the manufacturer’s instructions. After 48 hrs, the luciferase assay was performed using a Dual luciferase assay kit (Promega) and a single-sample luminometer (PerkinElmer, Waltham, MA). The luciferase activity was normalized by pSV-*Renilla* activity, and the pGL2-basic vector was used as a control. The pGL2-*CDO1*-Luciferase constructs and the pGL2-basic vector were methylated *in vitro* using *Sss*I (CpG) methylase as recommended by the manufacturer’s instructions (New England Biolabs, Beverly, MA). After DNA isolation, equal amounts (100 ng) of the methylated or unmethylated luciferase constructs were transfected into cells. Each experiment was performed twice, each in triplicate.

### Cancer Profiling Arrays Analysis

Cancer Profiling Arrays II with overall 19 different types of cancers (breast, kidney, rectum, colon, stomach, skin, thyroid, small intestine, bladder, vulva, pancreas, prostate, cervix, testis, lung, ovary, uterus, liver, trachea) was purchased from BD Clontech (San Jose, CA), and used to analyze the expression of the *CDO1* gene in normal and tumor tissues. The array was hybridized with the *CDO1* cDNA probe labeled with ^32^P-α-deoxycytidine triphosphate according to the manufacturer's protocol. Ubiquitin cDNA was used as a control.

### Immunohistochemistry

Tissue microarrays were purchased from US Biomax, Inc. (Rockville, MD). The arrays have sections (5 µm) of cancer tissues, adjacent tissues 1.5 cm away from tumor, and non-malignant normal colon and esophagus tissues; Multiple organ normal tissue array (#BN00011), Colon adenocarcinoma (combination of adjacent and normal) tissue arrays, (#BC05021 and #BC05022), Colon cancer and matched adjacent normal tissue array including TNM, clinical stage and pathology grade (#BC05118), Advanced colon tumor tissue array including TNM, clinical stage and pathology grade (#CO1922), Esophagus cancer with matched normal adjacent tissue array (#ES801) and Esophageal cancer progression tissue array (#ES804). The tissues were deparaffinized and incubated with anti-CDO1 rabbit polyclonal antibody (1∶250 dilution) (kindly provided from Dr. Nina Booken at Ruprecht Karl University of Heidelberg [21] at 4°C overnight. They were then incubated in broad spectrum secondary antibody purchased from DAKO (Carpinteria, CA) for 30 min. After washing the slides in PBS, tissue sections were stained with freshly prepared DAB chromogen solution (DAKO). We treated tissues with streptavidin and biotin (Invitrogen) for 20 min each to block endogenous biotin levels. Sections were counterstained in Mayer’s Hematoxyline.

### Plasmid Construction and Stable Cell Lines

Full-length *CDO1* was synthesized from total RNA extracted from the HEK293 cells by PCR using Platinum Pfx DNA polymerase (Invitrogen), and cloned into pcDNA3.1 expression vector using the pcDNA3.1 Directional TOPO Expression Kit (pcDNA3.1/V5-His Topo vector) as manufacturer’s protocol (Invitrogen). Plasmid sequence analysis was performed to confirm the fidelity of the *CDO1* insert (data not shown). HCT116 and DLD-1 cells were transfected using Fugene-6 (Roche, Basel, Switzerland) in OPTI-MEM (Invitrogen) as per the manufacturer’s instructions. Clones or pooled clones were selected for stable cell lines 2 weeks after transfected cells were grown in the presence of G418 (1 mg/ml). G418-resistant, empty vector-transfected cells were pooled for a control clone. The expression of CDO1 in the cell line was confirmed by RT-PCR or by western blotting with anti-V5 antibody (Invitrogen) after extraction of whole cell lysates.

### Cell Growth Assay

Cells were plated in a 12-well plate at a density of 2 ∼ 3 × 10^4^ cells per well and incubated at 37 °C. The tetrazolium-based cell viability (MTT) assay was performed every day for indicated days. Results were expressed as an absorbance at 570 nm wavelength or % of the control.

### Colony Focus Assay

Cells were plated in a 6-well plate at a density of 200 cells per well and incubated in the presence of G418 (1 mg/ml) for 2 weeks and stained with 0.4% crystal violet solution (MeOH/10% acetic acid, 3∶1). After air drying, colonies were photographed under the microscope and counted. Two independent experiments were performed and each experiment was done in triplicate.

### Soft Agar Assay

Cells (1×10^4^) were seeded in 1 ml of 0.3% low-melting agarose over a 0.6% agar bottom layer in McCoy, 5×supplemented with 10% FBS and 1 mg ml−1 of G418. The medium was changed three times a week and the clones were allowed to grow for 10 days. Two independent experiments were performed and each experiment was done in triplicate.

### Mouse Xenograft Assay

Athymic nude mice were divided into two groups (n = 5) and injected subcutaneously on the right flanks with DLD-1-p*CDO1*-#2 or DLD-1-p3.1 (5×10^6^ cells/200 µl PBS/flank) by using a 1-ml syringe fitted with a 27-gauge needle. Tumor size was documented by direct measurement in two directions by using Pro-Max calipers (Fowler Instruments, Newton, MA), and the measurements were recorded as tumor volumes (V, mm^3^). Tumor volume was calculated by the equation of *V = (L X S^2^)/2*, (L, long diameter, S, short diameter), and was measured once a week. All animals were maintained in accordance with the guidelines of the Johns Hopkins University Animal Care and Use Committee and the National Research Council.

### Chemoinvasion Assay

Cells were seeded at a density of 5×10^3^/well in transwell chamber (Lowell, MA) previously coated with Matrigel (upper part) and type I collagen (lower part). Cells were incubated for 16 hrs. A total of 10 sites per membrane were randomly selected for cell counting. Simultaneously, equal number of cells was seeded on 24-well plates, incubated for 16 hrs, and MTT assay was performed. Matrigel was purchased from BD Biosciences (San Jose, CA).

### Knockdown of *CDO1* and Cell Growth Assay

Four individual siRNAs targeting *CDO1* gene and nontargeting control siRNA were purchased from Dharmacon (Chicago, IL, USA). On-target plus set of 4 duplex (05 ∼ 08) was re-labeled as *CDO1* siRNA-1 ∼ -4, respectively. A 50 nM portion of each siRNA was transiently transfected to HEK293 or HepG2 cells using LipofectamineRNAiMax transfection reagent (Invitrogen) in OPTI-MEM. After 24 h, cells were incubated in complete growth medium. Initial cell seeding density was 3×10^3^ per well in 96-well plates for HEK293 and 2×10^4^ per well in 12-well plates for HepG2, and MTT assay was performed at indicated time points. For colony focus assay, HepG2 (1×10^4^ per well of 6-well plates) were transfected with siRNA and incubated for 8 days after addition of growth medium.

### Statistical Analysis

We used *CDO1* gene methylation levels (TaqMeth V) to construct receiver operating characteristic (ROC) curves for the detection of human cancer. In the ROC analysis, tangent points where the slopes of ROC curves were 1.00 have been selected as optimal cut-off points to balance sensitivity and specificity. P value was derived from Z value that was calculated from the equation of (AUROC-0.5)/Std Err (standard error of AUROC). The cut-off values determined from ROC curves were then applied to determine the frequency of gene methylation. Samples with a methylation level higher than cut-offs were designated as methylated, and samples with a methylation level lower than cut-offs were designated as unmethylated. For validation of *CDO1* expression differences between normal and tumor samples and also between tumor samples with and without metastases we applied Fisher exact test. *P*-values <0.05 were considered statistically significant. All Statistical analyses in this study were conducted using STATA Version 9 (STATA Inc., College Station, TX).

## Supporting Information

Figure S1 Analysis of CDO1 methylation in CRC. A, One dense CpG island (colored area) resides 430 bp upstream of the TSS in the promoter region of CDO1. Primers for bisulfite-sequencing (Seq-F and Seq-R) and TaqMan-MSP (TQM-F and TQM-R) were designed within the region which covered most of the CpG-rich region proximal to the TSS (∼ 400 bp) in the CDO1 promoter. F, forward; R, reverse. TSS, transcription start site. TQM-P (P), the probe for TaqMan-MSP. A total of 38 CGs were numbered from the first to the last CG in the sequences as indicated. CpG islands in the CDO1 promoter were searched by using the on-line accessible software Methprimer. B, DNA methylation of the CDO1 gene was observed in all CRC cell lines tested, but not in normal colon tissues. Closed square, methylation (M); open square, Unmethylation (U). C, Combined bisulfite-restriction analysis (COBRA) was performed in 10 pairs of matched CRC (PT) and colon normal tissues (PN) to examine the CDO1 methylation. After digestion of gel-eluted PCR products with BstU1, samples were loaded on a 10% acrylamide gel, stained with 1 X SYBR Green Gold (Invitrogen) and visualized under UV light. Multiple cleaved bands by BstU1 digestion were detected in PT samples (*), indicating the continued presence of protected CGCG sequences as a result of methylation. Due to tissue heterogeneity, methylated and unmethylated alleles co-exist so that uncleaved bands (arrow) can be seen. Mock digestion (without BstU1) of PT samples resulted in the same uncleaved band. L, 1 Kb Plus DNA ladder. No. indicates patient number. D, Representative bisulfite-sequencing results of HT29, and PT/PN samples derived from patient No.45. All guanines present after sequencing that are complementary to methyl cytosines on the opposite DNA strand. *, methylated CpGs maintained after bisulfite treatment.
(DOCX)

Figure S2 Immunohistochemical analysis of CDO1 in colon and esophagus cancer tissue array. A, The expression of CDO1 in non-malignant colon tissues. NN, patients without cancer. B, A group of samples were derived from a single patient consist of colon adenocarcinomas (AD), matched cancer adjacent normal appearing tissue (NAT) and matched cancer adjacent tissues (Adjacent). Patients were numbered arbitrarily (Pt1 ∼ Pt3). C, CDO1 expression in ESCC. PT, ESCC; PN, matched normal appearing tissues.
(DOCX)

Figure S3 The growth properties of cancer cell lines with or without forced expression of CDO1. A, The MTT assay was performed in HCT116 and DLD-1 cells after transient transfections with CDO1 expressing plasmids (pcDNA3.1-CDO1, pCDO1) or control empty plasmids (pcDNA3.1, p3.1) for three days. Data are presented as % of the control at day 1, and two independent experiments were done in triplicate. Values indicate means ± SD. *, P<0.05 in T-test. B, Colony focus assays were performed after transfection with pCDO1 or p3.1 in HCT116 and DLD-1 cells (left). Cells were incubated in the presence of G418 (1 mg/ml) for 10 days and stained with 0.4% crystal violet solution (MeOH/10% Acetic acid, 3∶1). After air-drying, colonies were photographed under a microscope (right). Values are expressed as means ± SD and are derived from experiments done in triplicate. C, Colony focus assays were performed after transfection with pCDO1 or p3.1 in KYSE30, MCF-7, NUGC3, and H1431 cells. Cells were incubated in the presence of G418 (0.5 ∼ 1 mg/ml) for two weeks. D, Establishment of clones stably expressing CDO1 or control clones. CDO1 mRNA levels were confirmed by RT-PCR and qRT-PCR (data not shown) and CDO1 protein levels were by western blot analysis using anti-CDO1 and anti-V5 antibodies (data not shown). E, The in vitro cell invasion assay was performed in clones stably expressing CDO1 or control clones. Cells were incubated for 16 hrs, and after fixation and staining, invading cells were counted at 100 X magnification (left). Cell growth for 16 hrs determined by MTT assay was not significant (right). Two independent experiments were done in triplicate, and values indicate means ± SD.
(DOCX)

Table S1 Primer sequences for CDO1.
(DOCX)
