# Supplementary figures and images for: Cysteine Dioxygenase 1 Is a Tumor Suppressor Gene Silenced by Promoter Methylation in Multiple Human Cancers
Source: PLoS One. 2012 Sep 27;7(9):e44951. doi: 10.1371/journal.pone.0044951 (PMC3459978; doi:10.1371/journal.pone.0044951)

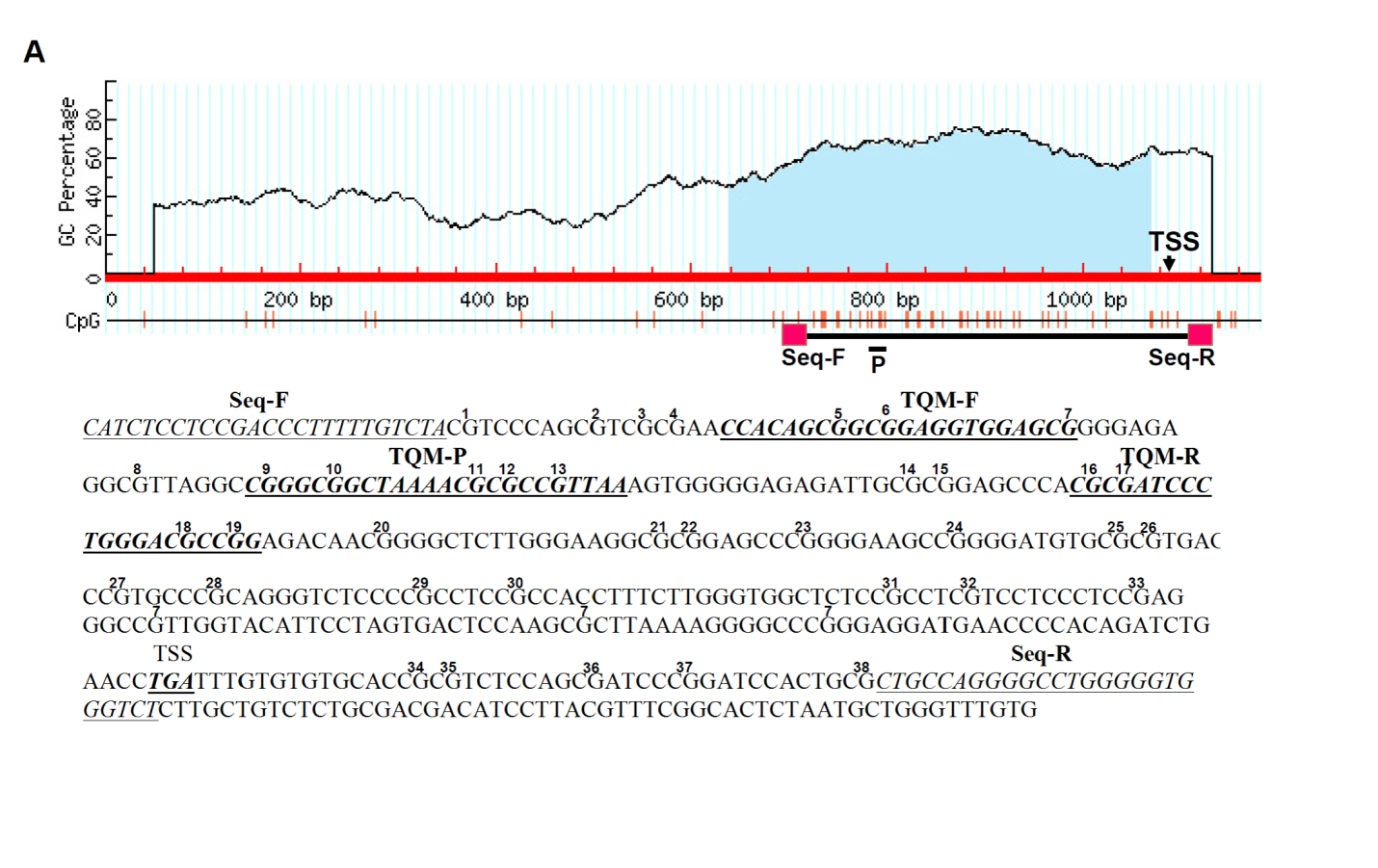


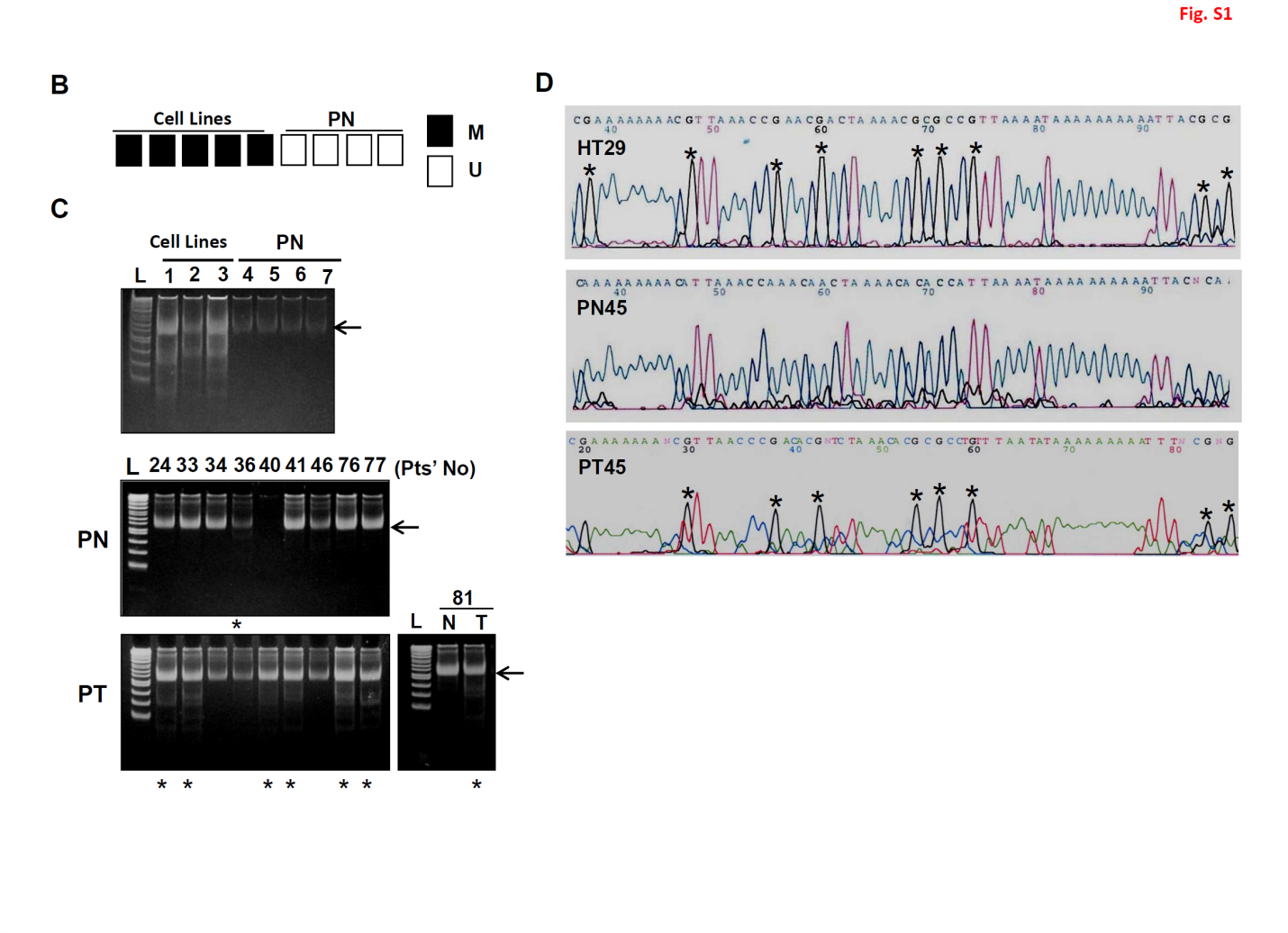

Supplement: Figure S1 — Analysis of CDO1 methylation in CRC. A, One dense CpG island (colored area) resides 430 bp upstream of the TSS in the promoter region of CDO1. Primers for bisulfite-sequencing (Seq-F and Seq-R) and TaqMan-MSP (TQM-F and TQM-R) were designed within the region which covered most of the CpG-rich region proximal to the TSS (∼ 400 bp) in the CDO1 promoter. F, forward; R, reverse. TSS, transcription start site. TQM-P (P), the probe for TaqMan-MSP. A total of 38 CGs were numbered from the first to the last CG in the sequences as indicated. CpG islands in the CDO1 promoter were searched by using the on-line accessible software Methprimer. B, DNA methylation of the CDO1 gene was observed in all CRC cell lines tested, but not in normal colon tissues. Closed square, methylation (M); open square, Unmethylation (U). C, Combined bisulfite-restriction analysis (COBRA) was performed in 10 pairs of matched CRC (PT) and colon normal tissues (PN) to examine the CDO1 methylation. After digestion of gel-eluted PCR products with BstU1, samples were loaded on a 10% acrylamide gel, stained with 1 X SYBR Green Gold (Invitrogen) and visualized under UV light. Multiple cleaved bands by BstU1 digestion were detected in PT samples (*), indicating the continued presence of protected CGCG sequences as a result of methylation. Due to tissue heterogeneity, methylated and unmethylated alleles co-exist so that uncleaved bands (arrow) can be seen. Mock digestion (without BstU1) of PT samples resulted in the same uncleaved band. L, 1 Kb Plus DNA ladder. No. indicates patient number. D, Representative bisulfite-sequencing results of HT29, and PT/PN samples derived from patient No.45. All guanines present after sequencing that are complementary to methyl cytosines on the opposite DNA strand. *, methylated CpGs maintained after bisulfite treatment. (DOCX) [file pone.0044951.s001.docx]

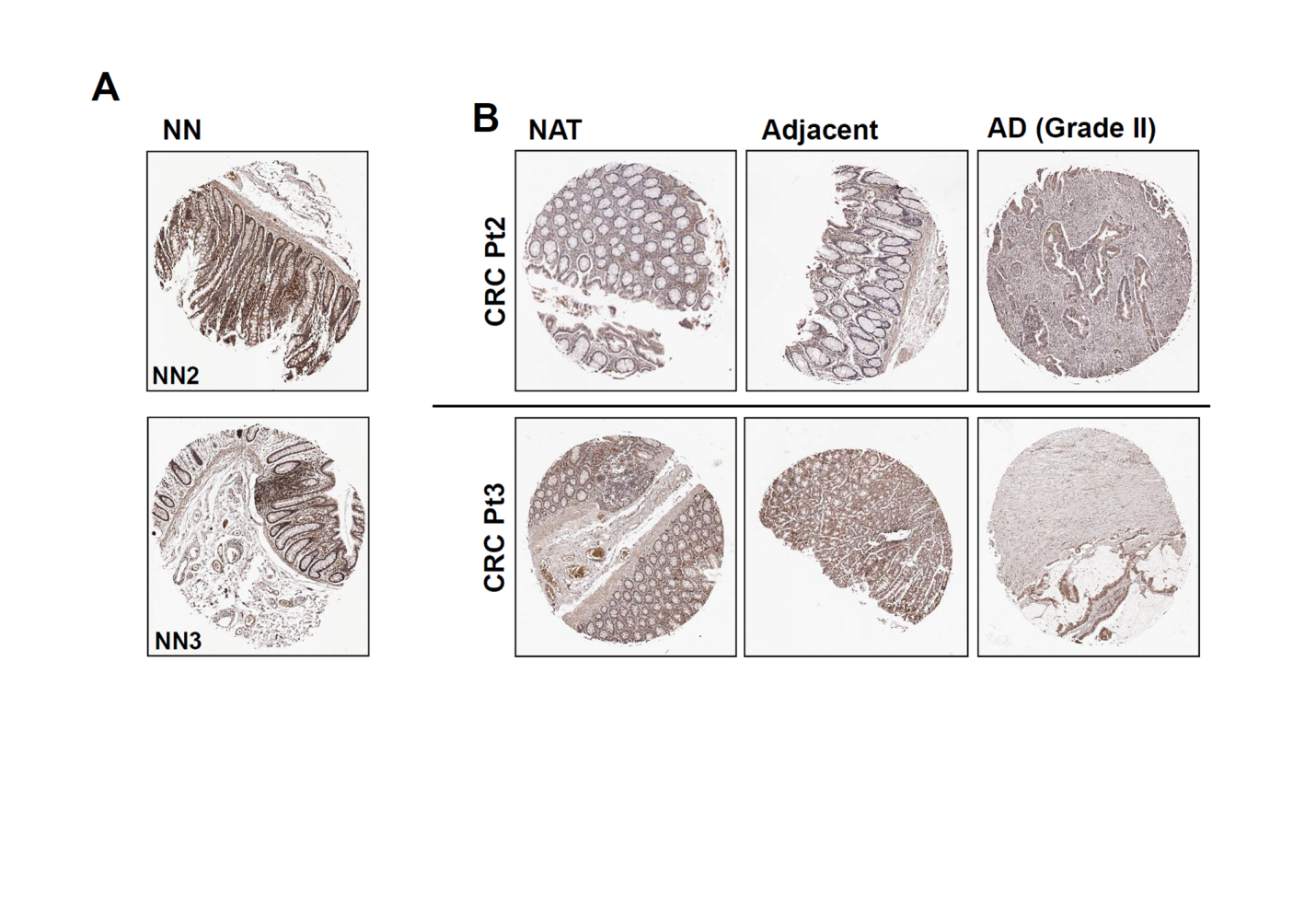


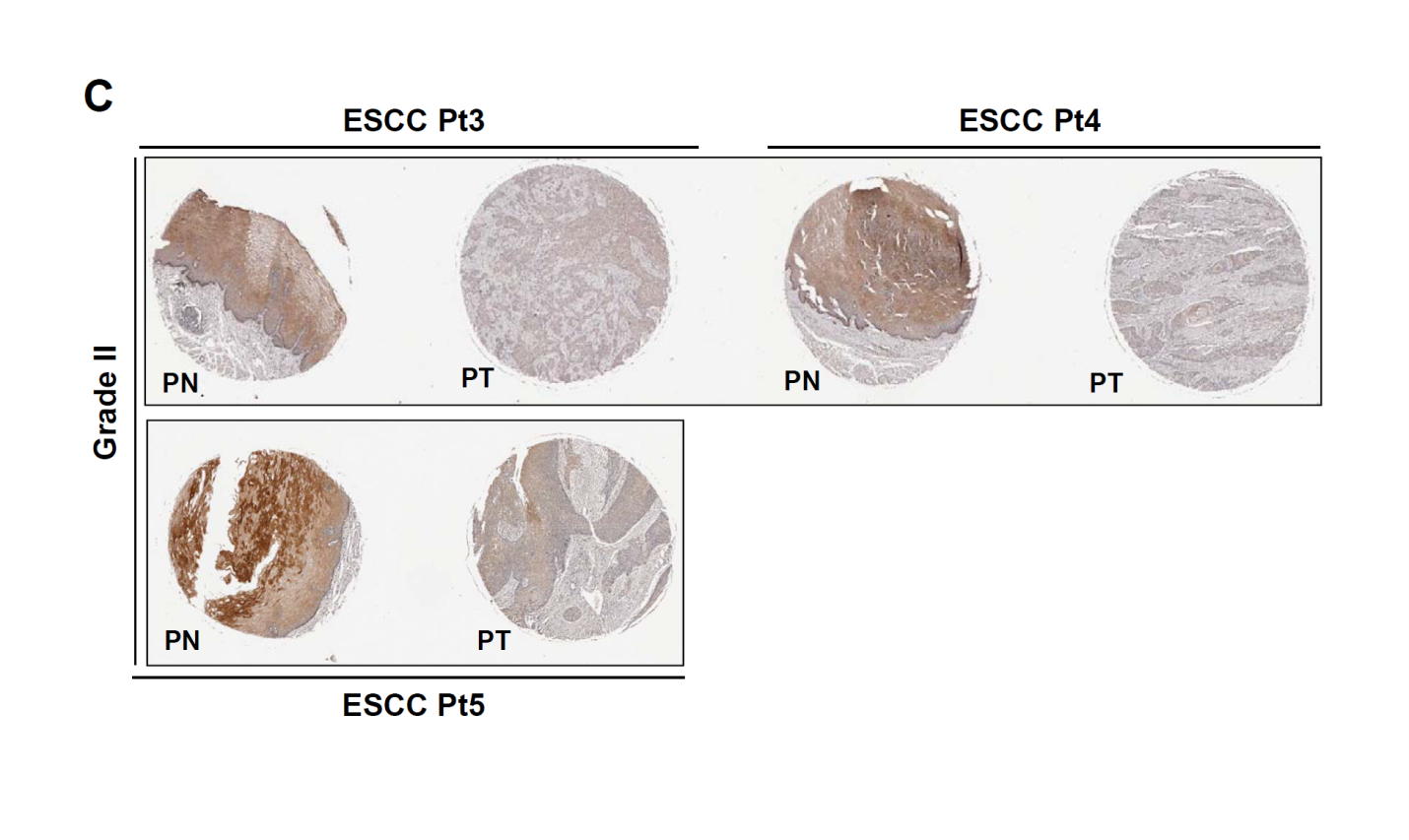

Supplement: Figure S2 — Immunohistochemical analysis of CDO1 in colon and esophagus cancer tissue array. A, The expression of CDO1 in non-malignant colon tissues. NN, patients without cancer. B, A group of samples were derived from a single patient consist of colon adenocarcinomas (AD), matched cancer adjacent normal appearing tissue (NAT) and matched cancer adjacent tissues (Adjacent). Patients were numbered arbitrarily (Pt1 ∼ Pt3). C, CDO1 expression in ESCC. PT, ESCC; PN, matched normal appearing tissues. (DOCX) [file pone.0044951.s002.docx]

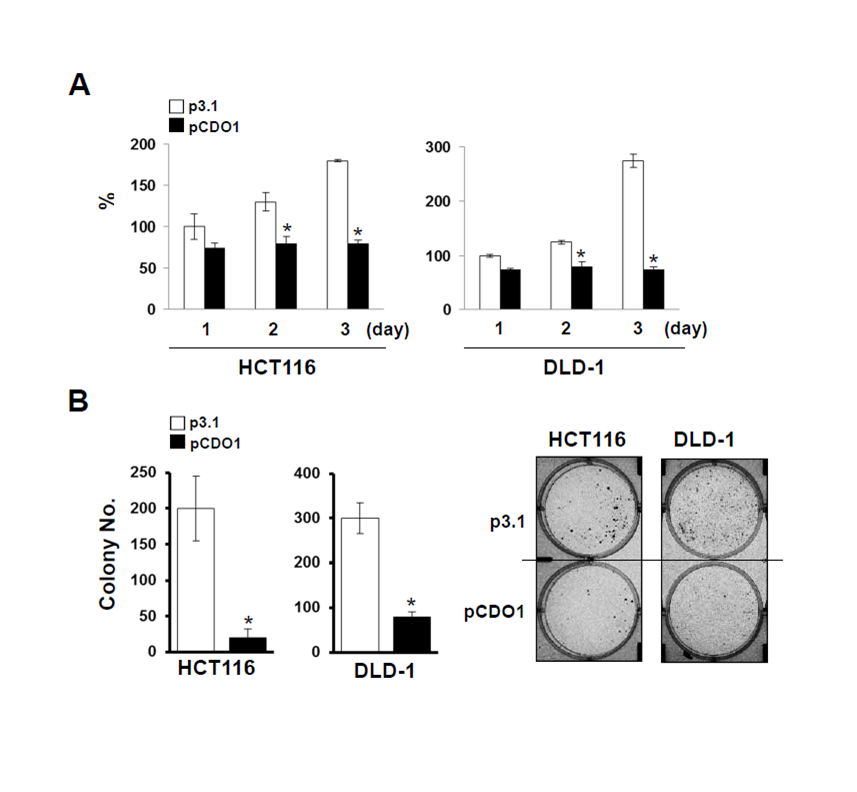


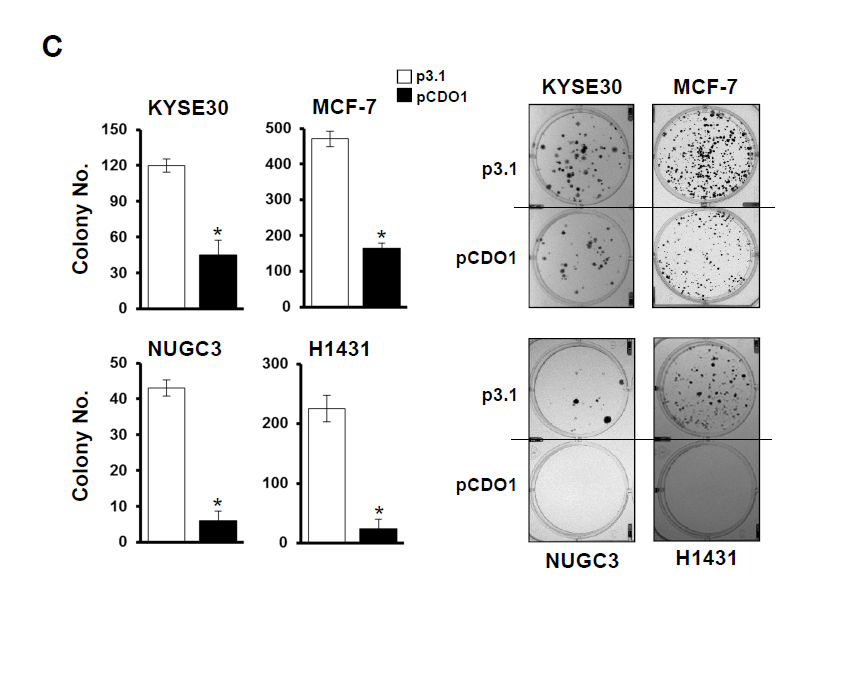


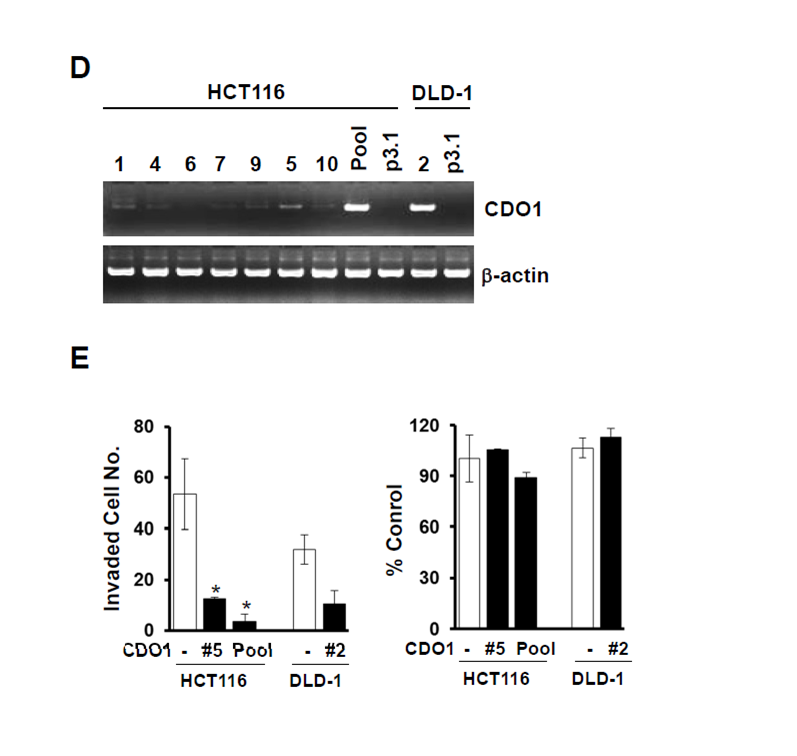

Supplement: Figure S3 — The growth properties of cancer cell lines with or without forced expression of CDO1. A, The MTT assay was performed in HCT116 and DLD-1 cells after transient transfections with CDO1 expressing plasmids (pcDNA3.1-CDO1, pCDO1) or control empty plasmids (pcDNA3.1, p3.1) for three days. Data are presented as % of the control at day 1, and two independent experiments were done in triplicate. Values indicate means ± SD. *, P<0.05 in T-test. B, Colony focus assays were performed after transfection with pCDO1 or p3.1 in HCT116 and DLD-1 cells (left). Cells were incubated in the presence of G418 (1 mg/ml) for 10 days and stained with 0.4% crystal violet solution (MeOH/10% Acetic acid, 3∶1). After air-drying, colonies were photographed under a microscope (right). Values are expressed as means ± SD and are derived from experiments done in triplicate. C, Colony focus assays were performed after transfection with pCDO1 or p3.1 in KYSE30, MCF-7, NUGC3, and H1431 cells. Cells were incubated in the presence of G418 (0.5 ∼ 1 mg/ml) for two weeks. D, Establishment of clones stably expressing CDO1 or control clones. CDO1 mRNA levels were confirmed by RT-PCR and qRT-PCR (data not shown) and CDO1 protein levels were by western blot analysis using anti-CDO1 and anti-V5 antibodies (data not shown). E, The in vitro cell invasion assay was performed in clones stably expressing CDO1 or control clones. Cells were incubated for 16 hrs, and after fixation and staining, invading cells were counted at 100 X magnification (left). Cell growth for 16 hrs determined by MTT assay was not significant (right). Two independent experiments were done in triplicate, and values indicate means ± SD. (DOCX) [file pone.0044951.s003.docx]

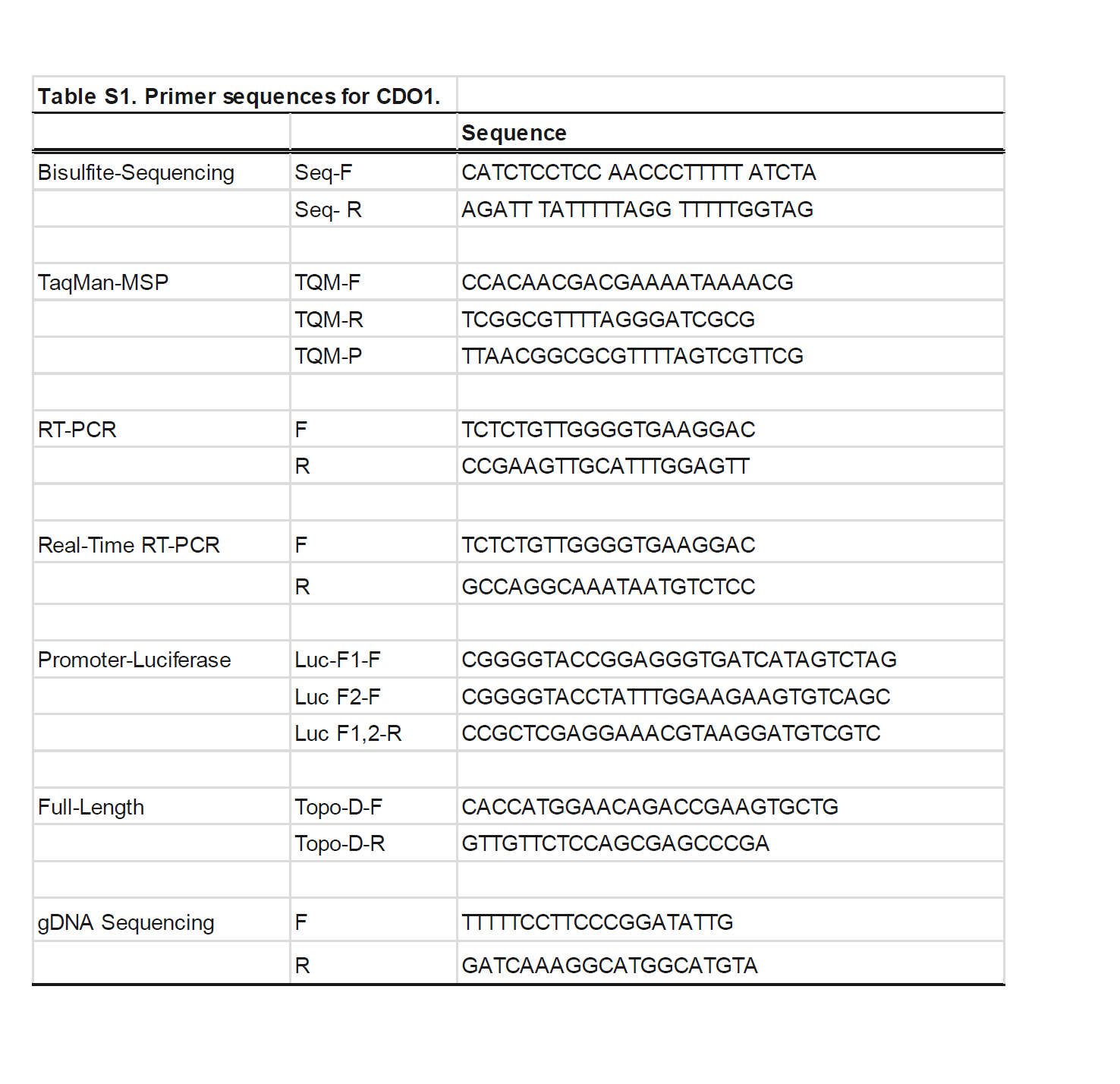

Supplement: Table S1 — Primer sequences for CDO1. (DOCX) [file pone.0044951.s004.docx]
